# aBravo Is a Novel *Aedes aegypti* Antiviral Protein That Interacts with, but Acts Independently of, the Exogenous siRNA Pathway Effector Dicer 2

**DOI:** 10.3390/v12070748

**Published:** 2020-07-11

**Authors:** Margus Varjak, Rommel J. Gestuveo, Richard Burchmore, Esther Schnettler, Alain Kohl

**Affiliations:** 1 MRC-University of Glasgow Centre for Virus Research, Glasgow G61 1QH, UK; r.gestuveo.1@research.gla.ac.uk; 2 Division of Biological Sciences, College of Arts and Sciences, University of the Philippines Visayas, Miagao, Iloilo 5023, Philippines; 3 Institute of Infection, Immunity and Inflammation, College of Medical, Veterinary and Life Sciences, University of Glasgow, Glasgow G12 8QQ, UK; richard.burchmore@glasgow.ac.uk; 4 Bernhard-Nocht-Institut for Tropical Medicine, 20359 Hamburg, Germany; schnettler@bnitm.de; 5 German Centre for Infection Research (DZIF), Partner Site Hamburg-Luebeck-Borstel-Riems, 20359 Hamburg, Germany; 6 Faculty of Mathematics, Informatics and Natural Sciences, University Hamburg, 20148 Hamburg, Germany

**Keywords:** mosquito, *Aedes aegypti*, arbovirus, antiviral response

## Abstract

Mosquitoes, such as *Aedes aegypti*, can transmit arboviruses to humans. The exogenous short interfering RNA (exo-siRNA) pathway plays a major antiviral role in controlling virus infection in mosquito cells. The Dicer 2 (Dcr2) nuclease is a key effector protein in this pathway, which cleaves viral double-stranded RNA into virus-derived siRNAs that are further loaded onto an effector called Argonaute 2 (Ago2), which as part of the multiprotein RNA-induced silencing complex (RISC) targets and cleaves viral RNA. In order to better understand the effector protein Dcr2, proteomics experiments were conducted to identify interacting cellular partners. We identified several known interacting partners including Ago2, as well as two novel and previously uncharacterized *Ae. aegypti* proteins. The role of these two proteins was further investigated, and their interactions with Dcr2 verified by co-immunoprecipitation. Interestingly, despite their ability to interact with Ago2 and Piwi4, neither of these proteins was found to affect exo-siRNA silencing in a reporter assay. However, one of these proteins, Q0IFK9, subsequently called aBravo (**a**edine **br**oadly active **a**nti**v**iral pr**o**tein), was found to mediate antiviral activity against positive strand RNA arboviruses. Intriguingly the presence of Dcr2 was not necessary for this effect, suggesting that this interacting antiviral effector may act as part of protein complexes with potentially separate antiviral activities.

## 1. Introduction

Mosquitoes transmit many medically and economically relevant arboviruses, including those belonging to the *Flaviviridae* and *Togaviridae* (genus Alphavirus) families, as well as those of the order *Bunyavirales* (previously *Bunyaviridae* family) [1,2,3,4,5,6,7,8].

Arboviruses actively replicate in mosquito cells, where immune responses counteract these processes. One of the most important antiviral defense mechanisms in mosquitoes is RNA interference (RNAi), which acts via sequence-specific target RNA breakdown mechanisms. There are two RNAi pathways in mosquitoes induced upon viral infection: the exogenous small interfering RNA (exo-siRNA) and the PIWI-interacting RNA (piRNA) pathways [9,10,11,12,13,14]. The exo-siRNA pathway is particularly important and has been shown to mediate antiviral activity. Viral RNA replication results in the synthesis of double-stranded RNA (dsRNA), which are targeted by the nuclease Dicer 2 (Dcr2) that slices these dsRNAs into mostly 21 nucleotide (nt) long virus-specific siRNA (vsiRNAs) in insects. Following this, vsiRNAs are loaded to the Argonaute 2 (Ago2) protein, which is part of the multiprotein RNA-induced silencing complex (RISC). One strand of the vsiRNA duplex is degraded and the remaining strand guides Ago2 to complementary viral RNA strand, resulting in its cleavage and degradation, assuming that mechanisms in mosquitoes resemble those of *Drosophila melanogaster* [15,16,17,18]. The production of vsiRNAs has been identified in arbovirus-infected mosquitoes, as well as in their derived cell lines for alpha-, flavi-, and bunyaviruses, and indeed Dcr2 and Ago2 can act antivirally against various arboviruses in mosquitoes or derived cell lines [9,11,12]. A possible exception might be Zika virus (ZIKV, *Flaviviridae*), which may at least to some extent be resistant to Ago2 activity, though it is still targeted by Dcr2 [19,20,21].

Virus-specific piRNAs (vpiRNAs) have been detected in mosquitoes and mosquito-derived cell lines following infection by viruses of all major arbovirus families/orders [10,12]. piRNAs are single-stranded and 24–29 nt in length and produced independently of Dicer activities. The primary role of piRNAs in most tested insects, is considered to be controlling transposons in germline cells. Most knowledge on the insect piRNA pathway is based on the *D. melanogaster* model, where piRNAs are produced in ovary follicular cells or in nurse cells; however, this is not applicable to mosquitoes [10,22,23,24,25,26]. Indeed, in mosquitoes, piRNAs are also produced in somatic cells and can be transposon, gene, or virus-specific. In addition, there has been an expansion of the PIWI protein family in mosquitoes, where *Ae. aegypti* has 8 (Piwi1-7 and Ago3) compared to the 3 PIWI proteins (Aub, Piwi, and Ago3) of *D. melanogaster* [27,28]. Although Piwi5, Piwi6, and Ago3 are mostly required for virus-derived piRNA production in mosquito, the only strongly antiviral PIWI protein in *Ae. aegypti* is Piwi4. However, its role in piRNA production and piRNA binding is not well understood and possibly disputable [19,29,30,31,32].

In general, there are gaps in our understanding of the regulation of the insect exo-siRNA pathway. Only recently, the Domino ortholog p400 was shown to regulate Ago2 levels in *Ae. aegypti* [33]. Neither are interactions of RNAi effectors with cellular partners characterized, bar a few exceptions. Protein interactions are often critical to understanding how and where an antiviral effector, such as Dcr2, acts, and, in the context of the exo-siRNA pathway, this therefore justifies further investigation. Indeed, Dcr2 is a major antiviral protein in mosquitoes, yet its interaction partners are not characterized in vector cells. Besides, there are likely differences from *D. melanogaster*. Indeed, studies so far have shown that, in the *D. melanogaster* siRNA pathway model, Dcr2 interacts with R2D2 and Loquacious (Loqs) proteins, which is required for linking steps from dsRNA cleavage to Ago2 loading, where Hsc70/Hsp90 chaperone machinery plays an important role [34,35,36,37,38,39,40,41,42,43,44,45]. Intriguingly, in *Ae. aegypti* Loqs2, paralogue of Loqacious and R2D2 is required for antiviral activity against dengue virus (DENV, *Flaviviridae*) in midguts [46].

The lack of data with regards to this pathway in *Ae. aegypti* has largely been due to lack of available tools, such as antibodies against mosquito proteins, expression systems, etc. Recent work by us and others have resulted in the establishment of novel tools to stably express proteins in aedine cells, including tagged proteins, which permits their capture.

In the current study, we used such a previously established cell line, an *Ae. aegypti*-derived Aag2 cell line that stably expresses *Ae. aegypti* Dcr2 [32] to identify interaction partners and thus potentially new antiviral proteins, given that Dcr2 plays a critical role in insect immunity.

Using a label-free quantitative proteomics approach, we identified two novel partners in addition to several RNAi-pathway members known in *D. melanogaster*: Ago2, Hsc70-3 (orthologue of human GRP78), Loquacious (LOQs), and R2D2. cDNA sequences were amplified, cloned, and expressed; the interaction between Dcr2 and the two novel partners was confirmed by co-immunoprecipitation. One of the proteins, which we called aBravo (**a**edine **br**oadly active **a**nti**v**iral pr**o**tein), was found to have antiviral properties against alphaviruses (Semliki Forest [SFV] and chikungunya [CHIKV]), as well as the flavivirus ZIKV. Interestingly, neither protein appeared to be involved in RNA silencing. This suggests that antiviral complexes in mosquito cells may consist of interacting proteins that carry out independent functions but may detect viral patterns within one complex.

## 2. Materials and Methods

### 2.1. Cells

*Ae. aegypti*-derived Aag2 cells (a kind gift of Paul Eggleston, Keele University, Keele, UK) were grown in L-15 medium plus Glutamax (Thermo Fisher Scientific, Waltham, MA, USA) supplemented with 10% tryptose phosphate broth (TPB; Thermo Fisher Scientific, Waltham, MA, USA), 10% fetal bovine serum (FBS; Thermo Fisher Scientific, Waltham, MA, USA), and penicillin–streptomycin (final concentrations of 100 units/mL and 100 µg/mL, respectively; Thermo Fisher Scientific, Waltham, MA, USA) at 28 °C. For Aag2-derived cell lines expressing V5-tagged proteins enhanced green fluorescent protein (eGFP), Dcr2, Ago2, and Piwi4 (Dcr2 GeneBank ID: AAW48725, Ago2 GeneBank ID: ACR56327 and Piwi4 sequence derived from Aag2 cells) [32], zeocin (Thermo Fisher Scientific, Waltham, MA, USA) was added to a final concentration of 100 µg/mL to main the expression of gene of interest. *Ae. aegypti*-derived Dcr2 KO cells (AF319) and the parental cell line AF5 [32] were grown in the same conditions as Aag2 cells. BHK-21 cells were used to propagate SFV and CHIKV and were grown in Glasgow minimum essential medium (Thermo Fisher Scientific, Waltham, MA, USA) supplemented with 10% TPB, 10% FBS, and penicillin–streptomycin (final concentrations of 100 units/mL and 100 µg/mL, respectively) at 37 °C, 5% CO_2_. A549-NPro cells (a kind gift of R. E. Randall, University of St. Andrews, St. Andrews, UK) were grown in Dulbecco’s Modified Eagle’s medium (DMEM; Thermo Fisher Scientific, Waltham, MA, USA), supplemented with 10% FBS, 2 µg/mL puromycin, penicillin-streptomycin (final concentration 100 units/mL, 100 µg/mL, respectively); to grow and titrate ZIKV, puromycin was left out [47].

### 2.2. Plasmids and Viruses

The firefly luciferase (*FFLuc*) and *Renilla* luciferase (Rluc) expression plasmids pIZ-Fluc and pAcIE1-Rluc, respectively, have been previously described [48]. The plasmid pPUb (with *Ae. aegypti* polyubiquitin promoter to direct gene expression) was used to generate the reminder of the expression constructs [32]. Target genes of interest from cDNA synthesized on total RNA isolated from Aag2 cells; a myc-tag sequence was added to the N-terminus of the protein during cloning. Plasmid pCMV-SFV4(3H)-*FFLu*c, contains reporter virus cDNA based on the SFV4 clone of SFV, virus SFV4(3H)-*FFLuc* expresses *FFLuc* as part of its non-structural polyprotein and is liberated by viral nsP2 [32,49]. Wild-type SFV4 was rescued from plasmid pCMV-SFV4 [50]. pSP6-ICRES1-2SG-*FFLuc* construct is based on LR2006OPY1 strain of CHIKV belonging to East/Central/South African genotype [31]; in this virus, *FFLuc* is expressed from duplicated subgenomic promoter. ZIKV strain PE243 used in this study was isolated in Brazil [51].

### 2.3. Protein Immunoprecipitation

Immunoprecipitation (IP) was carried out as previously described [32]. Briefly, 3 × 10^7^ cells stably expressing V5-eGFP or V5-Dcr2 were scraped and collected, followed by washing with phosphate-buffered saline (PBS). Thereafter, the cells were resuspended in lysis buffer (150 mM NaCl, 5 mM MgCl_2_, 20 mM HEPES (pH 7.4), 0.5% Triton X-100, protease inhibitor cocktail (Thermo Fisher Scientific, Waltham, MA, USA). Cells were kept on ice for 20 min, followed by centrifugation at 15,000× *g* at 4 °C for 25 min. The supernatant was then transferred into fresh tubes on ice, and mouse anti-V5 (1:500; AB27671; Abcam, Cambridge, UK) was added to the supernatant. Tubes were rotated for 2 h, at 4 °C. Following this, G-coated magnetic beads (Dynabead protein G; Thermo Fisher Scientific, Waltham, MA, USA) were added per sample after the beads had been equilibrated with cold washing buffer (150 mM NaCl, 5 mM MgCl_2_, 20 mM HEPES [pH 7.4], 0.5% Triton X-100). Tubes were then again rotated for 1 h, at 4 °C. The samples were washed 4 times with cold washing buffer. After the final wash, beads were resuspended in sample buffer (per 100 µL of buffer 25 µL of 4× Bolt lithium dodecyl sulfate sample buffer, 10 µL of 10× Bolt reducing agent (Thermo Fisher Scientific, Waltham, MA, USA) 65 µL of H_2_O) and boiled at 95 °C for 10 min. Protein samples were analyzed at Glasgow Polyomics on an LTQ-Orbitrap Elite instrument (Thermo Fisher Scientific, Waltham, MA, USA) coupled to UltiMate 3000 RSLCnano System chromatography system (Thermo Fisher Scientific, Waltham, MA, USA). Sample processing and analysis were carried out as previously described [47]. Interactors were identified using MaxQuant software and search conducted against UniProt database of *Ae. aegypti* proteins. For each protein, label-free quantification was carried out, and MaxQuant software-specific LFQ values were obtained; a protein was considered an interactor if present in samples with at least two peptides.

For co-immunoprecipitation, 10^7^ cells in a T75 flask stably expressing the protein of interest were transfected with 10 µg of plasmid expressing myc-tagged protein in pPUb using Dharmafect 2 reagent (Horizon Discovery, Cambridge, UK). At 48 h post-transfection (p.t.), immunoprecipitation was carried out as described above with the exception that mouse anti-myc antibody (1:500; catalog number 2276; Cell Signaling Technology, Danvers, MA, USA) was used.

### 2.4. Western Blot Analysis

Samples were run on Bolt 4–12% Bis-Tris Plus gels (Thermo Fisher Scientific, Waltham, MA, USA) and transferred to 0.45 µm nitrocellulose membrane (Thermo Fisher Scientific, Waltham, MA, USA) using a Trans-Blot SD Semi-Dry Transfer Cell (Bio-Rad, Hercules, CA, SA). Five percent (*w/v*) non-fat dry milk in Tween-PBS (PBS with 0.1% Tween 20) was used to block membranes for at least 1 h. Membranes were probed with mouse anti-myc tag antibody (2276; Cell Signaling Technology, Danvers, MA, USA) or mouse anti-V5 antibody (AB27671; Abcam, Cambridge, UK) in 2% (*w/v*) non-fat dry milk in Tween-PBS over-night at 4 °C. Following three Tween-PBS washes, the membranes were incubated with anti-mouse secondary antibody conjugated with horseradish peroxidase (A16072; Life Technologies, Inc.) or goat anti-mouse DyLight 800 (SA5-35521, Thermo Fisher Scientific, Waltham, MA, USA) in 2% (*w/v*) non-fat dry milk in Tween-PBS for 1 h. Membranes were again washed three times with Tween-PBS; horseradish peroxidase signal detected on Bio-Rad Chemidoc system (Bio-Rad, Hercules, CA, USA) using chemiluminescence detection reagents (Thermo Fisher Scientific, Waltham, MA, USA) or alternatively on Odyssey CLx (LI-COR Biosciences, Lincoln, NE, USA) to detect fluorescence.

### 2.5. cDNA Synthesis and qRT-PCR

Trizol (Thermo Fisher Scientific, Waltham, MA, USA) was used to isolate total cellular RNA. The RNA was used for cDNA synthesis using SuperScript III (Thermo Fisher Scientific, Waltham, MA, USA) and oligo(dT)15 primer (Promega, Madison, WI, USA) or random hexamer primer (Promega, Madison, WI, USA), depending on experiment. qRT-PCR for aedine genes or the housekeeping gene S7 was performed on cDNA produced using oligo(dT)15 primer by utilizing gene specific primers (Supplementary Table S1), SYBR green master mix (Thermo Fisher Scientific, Waltham, MA, USA), and an ABI7000 Fast system (Applied Biosystems, Thermo Fisher Scientific, Waltham, MA, USA) according to the manufacturer’s guidelines. Alternatively, samples produced using random hexamer primers were used to analyze ZIKV replication with virus specific primers and S7 primers for normalization. Results were analyzed using the ΔΔCT method.

### 2.6. Transfection of Nucleic Acids

All transfections were carried out using Dharmafect 2 reagent (Horizon Discovery, Cambridge, UK). To silence host transcripts and assess effect on virus replication, 1.5–1.7 × 10^5^ Aag2 cells per well were grown in 24-well plates, transfected with 300 ng of gene-specific dsRNAs per well, followed by virus infections at 24 h p.t. To silence a host gene in AF5 or AF319 cells by using siRNAs, 20 pmol of siRNAs (Horizon Discovery, Cambridge, UK) were used per well of 24-well plate (Supplementary Table S1). For RNAi reporter assays Aag2 cells were grown in 48-well plates and mock-infected or infected with SFV4 at a multiplicity of infection (MOI) of 10; 6 h later, 150 ng of dsRNA against eGFP or host targets were transfected using Dharmafect 2; at 48 h p.t., 50 ng of pIZ-Fluc and 50 ng of pAcIE1-Rluc were co-transfected with 0.25 ng of dsRNAs (*FFLuc* specific or LacZ specific) or 0.25 ng of ng siRNA (targeting *FFLuc* or Hygromycin B resistance gene as control [32]) using Dharmafect 2, and the *FFLuc* and Rluc activities were measured 24 h later.

### 2.7. dsRNA Production

Gene-specific primers flanked by T7 RNA polymerase promoter sequences (Supplementary Table S1) were used to amplify unique gene-specific fragments that were validated through Sanger sequencing. PCR products were then used for *in vitro* transcription using the RNAi Megascript kit (Thermo Fisher Scientific, Waltham, MA, USA) according to manufacturer’s instructions. The obtained RNAs were treated with Dnase1 and RNaseA and purified using columns provided with the kit. Previously described and verified dsRNAs were used for silencing of Ago2, Piwi4, eGFP, and LacZ [19,29,31,32,33,52,53]; alternatively, new silencing dsRNAs were developed for newly identified host proteins (see Supplementary Table S1).

### 2.8. Luciferase Assays

Aag2 cells were lysed with passive lysis buffer (Promega) and *FFLuc* activity measured by using the Luciferase Assay System (Promega, Madison, WI, USA) for SFV4(3H)-*FFLuc* and the Steady-Glo luciferase assay (Promega) for CHIKV-2SG-*FFLuc*. To measure both *FFLuc* and Rluc activities in RNAi sensor assays, the Dual Luciferase Reporter Assay System (Promega, Madison, WI, USA) was utilized. Luciferase activities were determined on a GloMax luminometer (Promega, Madison, WI, USA).

### 2.9. Statistical and Data Analyses

All luciferase data from three independent repeats were presented as mean with standard error relative to controls. All qRT-PCR CT values from four independent experiments were normalized to S7 housekeeping gene. Following the ΔΔCT method, normalized CT values were expressed as mean relative RNA levels to controls with standard error. If needed values were log-transformed to observe conditions for two-tailed Student’s *t*-test at 0.05 level of confidence. All statistical analyses were performed in GraphPad Prism v.7 (GraphPad Software, San Diego, CA, USA). BLAST algorithm at National Center for Biotechnology Information (NCBI) (https://blast.ncbi.nlm.nih.gov/Blast.cgi) was used to search for protein homologs with default parameters, last assessed on 18th May of 2020. Conserved Domain Database of NCBI (https://www.ncbi.nlm.nih.gov/Structure/cdd/wrpsb.cgi) was last used on 18th of May 2020 to identify conserved protein domains. Gene accession numbers are given as in VectorBase database (https://www.vectorbase.org/) with *Ae. aegypti* assembly AaegL5. Multiple sequence alignment (MSA) and phylogenetic tree was made using Geneious Prime v.2020.1.2 software (Biomatters, Ltd, Auckland, New Zealand). 

### 2.10. Data Availability

Data underlying luciferase and qRT-PCR figures are available here: http://dx.doi.org/10.5525/gla.researchdata.1010.

## 3. Results

### 3.1. Identification of Dcr2 Interactors and Antiviral Activities

Here, we used our previously developed Aag2 cell lines that stably express V5-tagged *Ae. aegypti* Dcr2 or enhanced green fluorescent protein (eGFP) [32] to conduct a proteomics study in order to identify cellular interaction partners of Dcr2. Samples from eGFP expressing cells were used as control, in order to identify the non-specific binders to magnetic beads and IP antibody. Those were used for subtracting, in the next step, to identify potential partners of Dcr2 and eliminate false positives.

**Figure 1:**
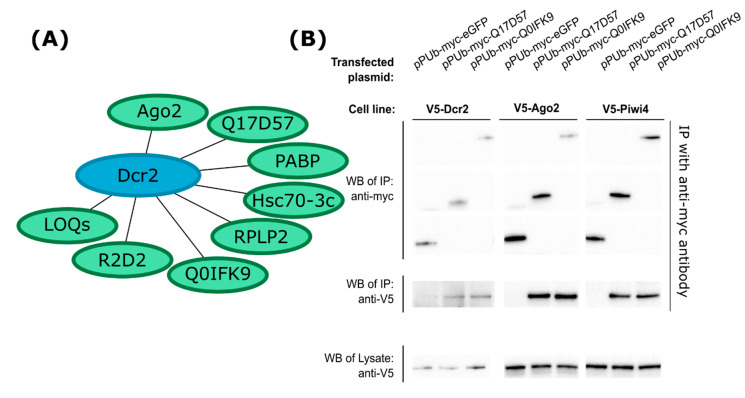
Summary of Dicer 2 (Dcr2) interactions and confirmation of interactions by immunoprecipitation (IP). (**A**) Protein associated (directly or indirectly) with Dcr2 from Aag2 cells; samples were obtained by IP followed by mass-spectrometry and database search. (**B**) V5-tagged protein-expressing Aag2 cell lines were transfected with myc-tagged protein expression constructs. At 48 h p.t., cell lysates were subjected to IP with anti-myc antibodies. In Western blot (WB) analysis, immunoprecipitated samples were probed for the presence of V5 and myc tags, and cell lysates were analyzed for V5 tagged proteins Dcr2, Ago2, and Piwi4; data shown are representative of three independent experiments. LOQs, Loquacious; PABP, poly(A)-binding protein; RPLP2, ribosomal protein lateral stalk subunit P2; Ago2, Argonaute 2; Dcr2, Dicer 2.

A protein was considered of interest if it was identified in at least 2 out of 3 replicates in Dcr2 IP samples, and no more than once in the eGFP control. For protein identification at least two peptides were needed (Supplementary Table S2 and summarized in Figure 1A). Among several proteins, we identified Ago2, Loqs protein, R2D2 and Hsc70-3. Their homologs in *D. melanogaster* that are known interaction partners of Dcr2 or otherwise involved in RNAi are discussed above. We also identified poly(A)-binding protein (PABP), ribosomal protein lateral stalk subunit P2 (RPLP2) and two previously uncharacterized *Ae. aegypti* proteins, with accession numbers Q17D57 and Q0IFK9 as per Uniprot database. BLAST algorithm search against the National Center for Biotechnology Information (NCBI) database indicated that neither of these proteins have well-characterized homologs in *D. melanogaster*.

Target gene mRNAs were amplified by PCR from cDNA, and we successfully cloned these genes into pPUb expression vector with the proteins tagged at the N-terminus with a myc-tag. Q0IFK9 was identical at the amino acid level to AAEL004699-PA in VectorBase, however, Q17D57 was found to have 6 amino acid differences compared to AAEL004317-PA in this database (for sequences, see Supplementary File S1). To verify their interaction with Dcr2, the Aag2 cell line expressing V5-tagged Dcr2 was transfected with expression constructs as indicated, followed by IP using myc-tag specific antibody. Unlike myc-tagged eGFP, myc-Q17D57 and myc-Q0IFK9 pulled down Dcr2, thus verifying the proteomics results (Figure 1B). Since Dcr2 is a major antiviral protein, we aimed to test whether these two novel proteins can interact with other antiviral proteins, as well. For this, we used V5-tagged Ago2- or V5-tagged Piwi4-expressing cell lines, and we indeed found that both aedine proteins interacted with Ago2 and Piwi4 (Figure 1B).

**Figure 2:**
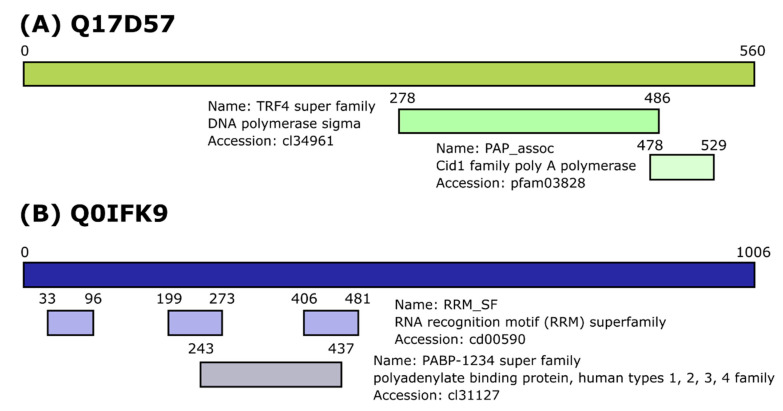
Description of novel *Aedes aegypti* Dcr2 interactors. Conserved protein domains based on National Center for Biotechnology Information (NCBI) Conserved Domain Database in Q17D57 (**A**) and Q0IFK9 (**B**).

Analysis for conserved protein domains, indicated that Q17D57 contains a TRF4 domain found in DNA polymerase sigma, and a nucleotidyltransferase domain of the poly(A) polymerase of Cid1 family (Figure 2). Cid1 proteins can regulate the production and degradation of microRNAs (miRNAs) [54,55]. It is therefore likely that Q17D57 is related to terminal uridylyltransferase protein Tailor found in the *D. melanogaster*, which has Cid1 domain, despite low sequence similarity [56]. Q0IKF9 has multiple RNA recognition motifs at the N-terminal half of the protein (Figure 2B), though no predictions could be made about potential functions.

Importantly, both proteins Q17D57 and Q0IFK9 appeared to lack homologs in *D. melanogaster*. The BLAST algorithm on NCBI server identified only one homolog for Q17D57 (with no known function) in *Ae. albopictus* mosquitoes. Q0IFK9 has homologs, also with no known functions, in *Ae. albopictus*, *Culex quinquefasciatus*, *Anopheles gambiae*, nor *An. sinensis* mosquitoes, which indicates conservation and potentially important role(s) in mosquitoes (phylogenetic tree, Supplementary Figure S1; multiple sequence alignment, Supplementary File S2; both produced by using Geneious software).

**Figure 3:**
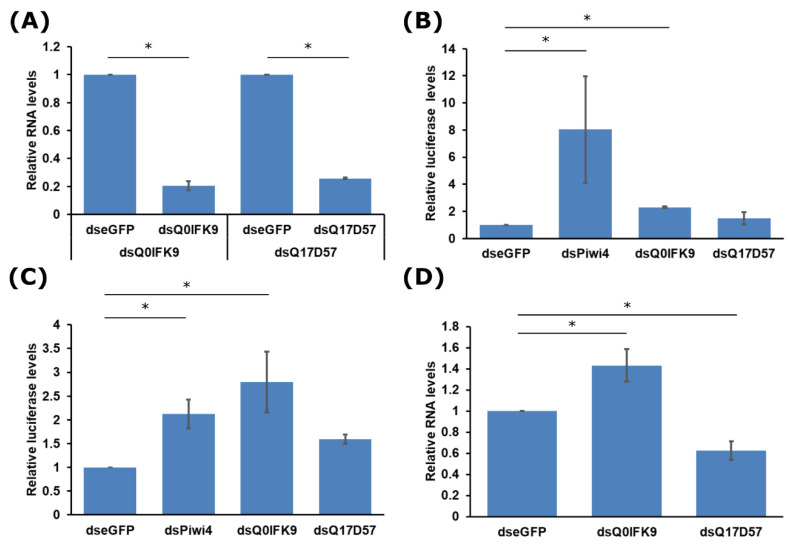
Effect of Q17D57and Q0IFK9 silencing of Semliki Forest (SFV), chikungunya (CHIKV), and Zika virus (ZIKV) replication. (**A**) Aag2 cells were transfected with double-stranded RNAs (dsRNAs) to silence transcripts encoding Q17D57, Q0IFK9, or enhanced green fluorescent protein (eGFP) as control; silencing efficiency was assessed by qRT-PCR on total RNA isolated 24 h p.t., using ΔΔC method and ribosomal S7 as housekeeping gene. The mean values of three independent experiments with standard error are shown. Transfected cells were infected with SFV(3H)-*FFluc* (**B**) or CHIKV-2SG-*FFLuc* (**C**) at multiplicity of infection (MOI) 0.01, 24 h p.t. and lysed at 48 h p.i.; *FFLuc* activities were determined and presented as relative mean luciferase activity values (normalized to activity in eGFP dsRNA treated control); the error of the mean from three independent experiments conducted in triplicate are shown. (**D**) Cells transfected as described above were infected with ZIKV PE243 at MOI 0.1 and total RNA isolated at 72 h p.i. to quantify viral RNA levels (S7 used as housekeeping gene). The mean values of four independent experiments with standard error are shown. For statistical analysis, values in (B) and (C) were log-transformed to reduce the heterogeneity; * indicates significance, *p* < 0.05 (Student’s two-tailed *t*-test).

Next, we tested whether Q17D57 and Q0IFK9 had any antiviral activity in the *Ae. aegypti*-derived Aag2 cell line. Cells were transfected with sequence-specific dsRNA to successfully silence Q17D57 or Q0IFK9 (Figure 3A). Infection with *FFLuc* expressing SFV at MOI 0.01 indicated that Q0IFK9 was antiviral as shown by higher levels of luciferase at 48 h post infection (h p.i.) following silencing, compared to eGFP-specific dsRNA control cells (Figure 3B). An increase in luciferase was also observed following silencing of Piwi4, as expected. Similarly, for CHIKV (MOI 0.01), higher luciferase levels were measured for Q0IFK9 and Piwi4 silenced cells (Figure 3C). The effects of silencing of Piwi4 or Q0IFK9 on SFV or CHIKV cannot be compared directly; for the former, the luciferase reporter is expressed as a part of non-structural polyprotein and liberated by virus nsp2 proteolytic cleavage, and, in the case of latter, the reporter is expressed under the control of a duplicated subgenomic promoter. As the mode of action of Q0IFK9 is not known, the amplitude of effect(s) on viral replication may be affected by the position of the reporter gene. For ZIKV infection (MOI 0.1), a small but significant increase in viral RNA levels was measured by qRT-PCR at 72 h p.i. after Q0IFK9 silencing compared to control cells (Figure 3D). Taken together, these results indicate that Q0IFK9 acts as an antiviral protein against these positive strand RNA viruses. No increase in luciferase (for SFV or CHIKV) nor RNA levels (ZIKV) RNA was observed in Q17D57 silenced cells compared to control cells (Figure 3B–D).

### 3.2. Effects of Dcr2 Interactors on the Exo-siRNA Pathway

**Figure 4:**
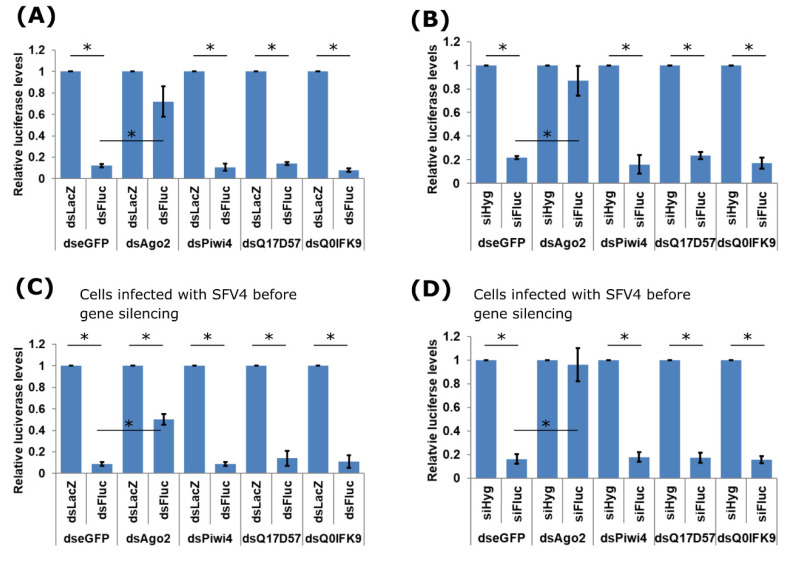
Effect of Q17D57 and Q0IFK9 knock down on the exo-siRNA pathway. Aag2 cells were transfected with (**A**, **B**) dsRNA targeting eGFP (control), Ago2, Piwi4, Q17D57 or Q0IFK9, or (**C**,**D**) first infected with SFV4 at MOI 10 followed by dsRNA transfection 6 h later. After 48 h, cells were co-transfected with *FFLuc* (pIZ-Fluc) and Rluc (pAcIE1-Rluc) expressing reporter plasmids and (**A**,**C**) dsRNAs against *FFLuc* (dsFluc) or LacZ (dsLacZ) (as control) or alternatively, (**C**,**D**) small interfering RNAs (siRNAs) targeting *FFLuc* (siFluc) or Hygromycin B resistance gene (siHyg) (as control) to assess the silencing ability of siRNAs and bypass the necessity for Dcr2 processing. Cells were lysed at 24 h p.t., and luciferase activities determined. Relative luciferase levels are shown on the Y-axis (with *FFLuc*/Rluc ratio in dsLacZ or siHyg transfected cells set to 1). The mean values with standard error are shown for three independent experiment conducted in triplicate, * indicates significance, *p* < 0.05 (Student’s two-tailed *t*-test).

The antiviral action of Q0IFK9 might be exerted via the exo-siRNA pathway, given the interactions with Dcr2 and Ago2. Hence, we next assessed if knock down of Q0IFK9 had an effect in a reporter assay, which is based on inducing the exo-siRNA pathway. Ago2, Piwi4, Q17D57, Q0IFK9 expression was silenced in Aag2 cells, followed by transfection of *FFLuc* and Rluc expressing plasmids together with dsRNA targeting *FFluc* (or LacZ as control; dsFluc or dsLacZ, respectively). In transfected cells, dsRNA is processed into siRNAs by Dcr2, then loaded to Ago2 followed cleavage of target RNA. As expected, in cells where Ago2 had been knocked down, silencing of *FFLuc* by specific dsRNAs was significantly hampered (Figure 4A). As Piwi4 is not connected directly to the siRNA pathway, as expected knock down had no effect on dsRNA-based luciferase silencing. Knock down of neither Q17D57 nor Q0IFK9 affected the dsRNA-based silencing efficiency on *FFluc* (Figure 4A).

Similar results were obtained if siRNAs instead of dsRNAs were used. No effect on silencing was detected if Piwi4, Q17D57 or Q0IFK9 had been silenced (Figure 4B), unlike Ago2. We also tested if the presence of replicating virus could affect the functioning of Q17D57 or Q0IFK9. The presence of SFV4 (MOI 10) did not affect the reporter assay (Figure 4C,D); as previously observed in uninfected cells, the knock down of Ago2 had a substantial effect on the exo-siRNA machinery.

**Figure 5:**
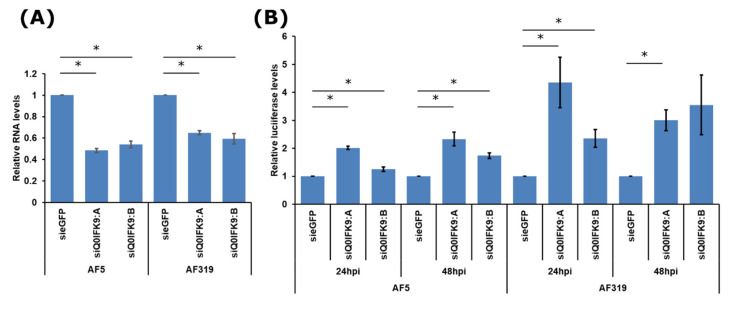
Effects of Q17D57and Q0IFK9 knock down on SFV replication in Dcr2 knockout cells. (**A**) AF5 and AF319 (Dcr2 KO) cells were transfected with two different siRNAs against Q0IFK9, or siRNA against eGFP as control; knock down efficiency was assessed at 24 h p.t. by qRT-PCR with S7 as housekeeping gene. The mean values of four independent experiments with standard error are shown. (**B**) At 24 h p.t. with siRNAs as shown, AF5 or AF319 cells were infected with SFV(3H)-*FFLuc* at MOI 0.01 and luciferase levels were assessed 24 and 48 h p.i., for each cell line. Luciferase values of sieGFP treated cells was taken as 1 per time point for each cell line; the standard error of the mean from three experiments conducted in triplicate are shown. For statistical analysis, to reduce heterogeneity, log-transformed values in (**B**) were used; * indicates significance, *p* < 0.05 (Student’s two-tailed *t*-test).

These findings suggested that antiviral functions of Q0IFK9 were not connected to the exo-siRNA pathway. In order to investigate this further, experiments were conducted in Dcr2 knock out cell lines AF319, or the parental cell line AF5 which is derived from Aag2 cells [32,57]. dsRNAs cannot be used in AF319 cells due to the absence of Dcr2; thus, two different siRNAs were designed to knock down of Q0IFK9 in AF319 and AF5 cells (Figure 5A). Infection of the cells with SFV(3H)-*FFLuc,* showed that silencing of Q0IFK9 in AF5 cells resulted in increased virus replication, as measured at 24 and 48 h p.i. compared to control cells (Figure 5B). This is similar to results obtained with dsRNAs in Aag2 cells (Figure 3B). Furthermore, a similar effect was found to occur in AF319 cells at both time points and with both Q0IFK9 specific siRNAs, which suggests that Q0IFK9-mediated antiviral activity is independent of the presence of Dcr2.

## 4. Discussion

The exo-siRNA pathway is very important in the mosquito antiviral defense, and Dcr2 is a key effector at the first step of this pathway. However, a majority of the Dcr2 functional studies have been carried out in *D. melanogaster* and not in mosquitoes. For *D. melanogaster*, Dcr2 has several interaction partners whose functions have been described (i.e., Ago2, R2D2, Loqs; the two latter having a critical paralogue in *Ae. aegypti* missing in the midgut) in the siRNA pathway [34,35,36,37,38,39,40,42,43,44,45,46]. However, little is known about mosquito proteins that interact with this antiviral protein and its possible subsequent role(s) in processes, such as cutting dsRNAs into siRNAs and their transfer to Ago2.

Immunoprecipitation followed by mass spectrometry identified interaction partners of *Ae. aegypti* Dcr2 in cells derived from this mosquito, to the best of our knowledge this the first study of this kind. Among the captured proteins were Loqs and R2D2, as expected from work on *D. melanogaster*. Interestingly, two of the identified proteins (Q17D57 and Q0IFK9) had been predicted to be expressed in *Ae. aegypti* cells but not *D. melanogaster*. Both proteins were cloned, expressed, and shown to co-immunoprecipitate with Dcr2, confirming their interactions. Interestingly they also interacted with other antiviral RNAi proteins, Ago2 and Piwi4. However, it is not known whether these interactions are direct or indirect. The interaction network between various RNAi pathways in mosquito cells is being untangled at the moment. For example, Piwi4 can interact with Ago2 and Dcr2, as well as with proteins of the piRNA pathway, like Ago3 [32]. This suggests that different RNAi effectors complexes are present in cells, that might have different functions. It remains to be elucidated in future studies how the interactions between Q17D57, Q0IFK9, Dcr2, Ago2, and Piwi4 occur directly or indirectly, for example, via RNA bridges. Importantly, through our exo-siRNA reporter assay, we indicated that none of the newly identified proteins had a role in facilitating gene expression silencing via dsRNAs/siRNAs.

The C-terminal part of Q17D57 is similar to *D. melanogaster* terminal uridylyltransferase protein Tailor, which carries out uridylation of miRNAs that results in their degradation [56]. However, the N-terminus of protein Q17D57 has no sequence elements with known functional importance, and the full-length protein was found to be conserved only in *Aedes* mosquitoes. Thus, it is likely that Q17D57 has similar function to Tailor, which could be a topic for further studies. Alternatively, in *D. melanogaster* polyadenylation of Toll and R2D2 mRNA happens via Dcr2-dependent mechanism, as Dcr2 interacts with cytoplasmic poly(A) polymerase Wispy [58]. Considering that Q17D57 has a conserved nucleotidyltransferase domain of poly(A) polymerases, it might be that *Ae. aegypti* Dcr2 could have similar function in recognizing mRNAs and directing their polyadenylation. As we are not sure about the function, we propose at this stage to name this protein aDiuf (**a**edine **D**cr2 **i**nteractor of **u**nknown **f**unction).

Q0IFK9 has multiple RNA recognizing domains in its N-terminal half; however, there are no conserved domains with known function in the C-terminal half. It was found to be antiviral against alphaviruses, SFV and CHIKV, and the flavivirus ZIKV. Silencing did not negatively affect dsRNA-mediated silencing of gene-expression, and its antiviral activity was found to be independent of Dcr2. As Q0IFK9 interacts with Piwi4, we cannot exclude that it may act together with Piwi4 in a complex that has antiviral activities. However, in general, the antiviral role of piRNAs is unclear. The production of piRNAs that is dependent on Ago3, Piwi5 but less on Piwi6, is not needed for the antiviral activity of Piwi4 [29,31,32]. In infected cells, viral RNA can be converted into vDNA by transposon reverse transcriptases and Piwi4 binds piRNAs that are derived from these vDNA [30,59]. However, it needs to be determined if Q0IFK9 has a role in this process. This protein may also act independently of any of those pathways. More work is needed to determine the mechanism(s) of Q0IFK9 antiviral activity, but we believe that the data demonstrate that the various antiviral proteins interact at least at some point as part of multiprotein complexes that mediate antiviral activities. Given Q0IFK9 action against alphaviruses and a flavivirus, we propose to name this protein aBravo (**a**edine **br**oadly active **a**nti**v**iral pr**o**tein). Future experiments should investigate the effect(s) of aBravo in vivo, and its mode of action at molecular level. This could include identifying interaction partners of aBravo by employing proteomics tools, but also RNA binding partners by using approaches, such as CLIP-seq. Moreover, its cellular localization during viral replication will need to be investigated.

In summary, our proteomics-based approach revealed a novel effector in *Ae. aegypti* antiviral immunity. The tools generated will allow further studies on this protein and mode(s) of action.

## Supplementary Materials

The following are available online at https://www.mdpi.com/1999-4915/12/7/748/s1, Supplementary Figure S1: Phylogenetic tree *of Ae. aegypti* Q0IFK9 homologs across mosquito species. Supplementary Table S1: Primer and siRNA sequences. Supplementary Table S2: Summary of proteins identified by mass-spectrometry. Supplementary File S1: Q17D57 and Q0IFK9 gene sequences from Aag2 cells. Supplementary File S2: Multiple Sequence Alignment of Q0IFK9 homologs in other mosquito species.
