# Supplementary material for: aBravo Is a Novel Aedes aegypti Antiviral Protein That Interacts with, but Acts Independently of, the Exogenous siRNA Pathway Effector Dicer 2
Source: Viruses. 2020 Jul 11;12(7):748. doi: 10.3390/v12070748 (PMC7411624; doi:10.3390/v12070748)
Supplement: Supplementary file 1 [file viruses-12-00748-s001.zip › Supplementary File S1.pdf]

>Q17D57 gene sequence from Aag2 cells
ATGTCCCATTTCCACCTACAACTGTTCGCCTTTCGGCTCAATGAGATTATCGATGACTATCTCCAGCGAGTCAAC
TGGATCTACGAGGGCGCCGTCCCGGAGATCGATCACCTGTTGGAGGCGATCGATTCCACtCTGTTCGAAGCAGCA
TGTGTCCAGCCCGCAATCGCTGGTATTCGGAGATATTCAGTCCTGTCGGGACATTGTGCGGTTTTTTGAACCGAT
CCAGTGCTGTGGGAAAAGCCTCTTGAACAGCGACACGCTGATGGTGCACTTGTATGCATCACGCCGCCGATGAAC
GGGAAGAACTGGACAGTGGCACTAAGCGCACGGCCATTCTCTTCAAtCTTGTTCGTCCAACTTGGCACGGGTATT
CTTGACCGCTACGACAACAACTACGACCAGATTGAGCAATACACCGCTCAACTGCTGCGCAAGGCATCCGAATAC
CTAAGCTGCATTCTGCGGCAAGCCGATCCGAGGAGATGCCCtAGGCTGCGGTCACCTTCATCGAGAATGTCACCT
TTGCCAAGTGGAAGTACGTGTTCGACTACACGTGCATCCCGTGTACCAACAGGGGAATCGACAACTGCCGCTACG
AGGATGCAATCGCATGGTACAAAATCCACCTCAACACGATGGGGTCCAACCCGATTCGCCACCTGGGCACCAAGA
TGCATGGAGTAAGCCTTCGGGATTGTTTGGGGGATCGCGTTGTTGCACCAACTCCTTCGGTGATTCGGGAGGTTC
GACCAAGTCCCCAGCTgCTCCACGGCTACCAAaGTACCTCATGGAGGTCCGTGAAAGCGAGCTGGACAAGCTTTG
CACGATCGGAACGGCAGTTCAACGGAACACGCCTTACCAAGCCGTCAGAGCTTATCTTCAGGCGAAACTAGGATC
TATTCTCAAGAGGAGCACCATCGAACCATTCGGCTCGCGAGTAACTGCgAGTAGCCAATCAGAATTCGGATCTTG
ATCTGGCCATCAAAGTGGATCCTAGCATGCAACCGAAAACTGCCTACCAAAAAGTTCTTGCATGGGCCAGAGCAA
ACaGATCAtGGAAGTGcAATGTAGAACGTCAGATCCCTGACGGACCTTTGGTTCTTCGCTTGCAGATTCATTCCC
TGCAATTGATCGTGGACCTTACGTTCAACAGCCCATACGTGGTTGGTAATGCAAAGATCATTGACTACTTCTACC
GACTTCAACCGATGGCCCGCAAACTATTCTTCTTGCTCAAGGAATGGAAGAAGCGAACTGATATCGGCAAGAACT
TaCCACCACAATGTCCTAACTTCCATGATCGTATTCTACATGCAAACGGAACAGTATCTTCCCCCAATCACTCCG
CTGATCAACGGTCCCCAAAAGGTCAACGCCTTATACAACACGGAATTCGTTGAGCTCATCACCCTGTATCGACTT
CCGAACAGCTTTCTGGCCCTCGTAAAAGACTTCTTCACCTATTGGTCTCGCTTCGATTGGGCCCGTACCGGTGCA
TCGGTCAAGGACGCCAGAGTCCGACCCAGAAGCACCTTCCGACAATGTGCGCAACCCGACCCAGCCGATGATGAT
AACGGATTATTTCGACGTTACTCGCAACGTGGCCGGAAATGTCGCCCCAGGGGACTGCGAGAAGTTTGTCCGGGC
CTGCAAAGAGGCAGCAGGCGCTCTGAAGGCCAAACAGACCATCTGA

>Q0IFK9 gene sequence from Aag2 cells
ATGTCTGAAACCGAGAAGAAAGCAGAACAATCTACCGGAGGAGAAACCGAGACCGACGGCCCAAAGCCTGATGCTGAGCATCA
ACGCAAGATCAATGTCGCTAACTTGCCGTGGGACGTAACTGAGCAGCAGTTGCGCGATTTGTTCAGCGGTCATGAGGTCGAGA
AGGTGGAGATCTACCATTACCGCAAGTACACGCTGGCCTTGGTCCTGTTCAAGGACAAGGACACCGCTGCCGCGGCTCTgGCC
GAAAAGGAGAACTCTATGTTCCGCGATCGCCGTCTTCGTATGCACATGGAATTCACCTCAATCCGCCATATCAAGCGTGAAGT
CATTGTTACCGTCGTTGAAGATGAGAACATGACCGAAGAGCAGCTGTGGGATAAGGTTAAGGCAGCTGGCGTGGAACCGACTT
CGGTGTTGATCTTCTATCCATTGGGCTATGTTACTTTGGAGAAGGAATGCGACAAGGCcCCTATTCTTGAGAAACTGAAGGCC
GCTGGATTCAATGCGCATGACTTGAACCAACGTGACCAGAACCAGCATTTGGACATCTGGCGTGCGGCCAAACTGTTCTTCCG
TAACCGCAACCGtGTCCAGCTGTTGAACATTCCCAACAAGTGGATTGAAGATAAGGAAGAGATGAAGAAACAATGCGCTGAAG
GAGGTACCATTACCGAAGCTGTTTCGAACAATGTTACCCAAAATCCCCAGATGGCTTCCAACTATGCCCGAATCTTCTACGAA
ACTGAAGAGCAGGCCGTCAAGGCTGCAAAGGCTCTGAATGGACGCGTTGTTGATGGCAAGCGTATCCATGCTCTGCATCTGGG
ATCCGCATTGATGCCCAACTACAAGACCTCGGTCTACGTTACGTCTTTGGACAAGGGTGTTACTGAAGAGCAAGTTTATGATC
ATTTCAAGCAGTTTGGCGAAATTGAGTTCACCAACCGTCGCAACTGTGGAGATGCTGCTATCATCTGCTACAAGAACGCTGCT
TCCGCTGAGAAAGCGCTTGCATGCACCACCTTCCCGGCGCCTACCGAAGAGAACAAGGATGCTACCAAGGAGGTTGTTGTCAA
GAAATACGACGGACCATTGGTCCTTCGTCCGGTTGCCCTGAAGCGTAAATTGAAGGCCGGAGAAGAAGAGAAGGATCAGGCCG
AAAAGACCAAGGAGCAACAAAGTGTGCTCGGAAAGCTGAAGGCTTACCACCCGATCTTTGTCGGCAACGTGCCACTGAATTGC
CCACCTTTGGAATTGAAGCACTACTTTGCGCAGCACGGACCAATCAAGTTCATGTTCTCGCCGCAAATCTTGGCTTACCGCAA
CAGCGCGCCTCACCCCGTGAAGACGTTCATCATCTACTACATGAACCGTCCGAGCCAGCAGAATGCCGTTCGCTTCTTCGACC
ACAAGTTCTTCGGtGGTCACCGTCTGCATGTGCTGCCACTGCGCGGAACACAAAACATGGATGTCAAGAAGACCATCAAGATG
ACCAAGATTCCATTCATGTCCGAGGATGCGTTGTTCAAGAAGATCAAGCCATTCGTTGGCAAGATCAACCGCATCGTGAAGAA
GTCGCGATTGATAGCTTACATTGAGCTGGAAGACAGCGCCGACGTCGAGAAAATGATGAACGTTGAAACCCCTGACCATCCTC
TGCCAAAGGGTAAGATGGAACAGCTCAAGACCCCAATCAGCGCTCGTCTGTACAATGAGGACGATGTTCGTGTATTGAACGGC
ATCGCCAAGATTGTCGCCAAGAACCCGGATATGCTGAAGAAGAACATTCAGCCACCCAACAAGCGTCCACGCATGGAGTTCGG
TGGTCCAGGTTTTAATCAAGGCCCTGGAGGCTTCGTTCCCAATAACAATATGAACAATCCAAATGCTATCCAAGATTTGTTGC
GTCTTGCGTTCATGTCCGGCAAGAACGTTGGCGAGAGTCTGGCTTCTAACCAGAGCTCGTTCGGACCCGACCCACCAGTCGCC
AATTCCAACAACTTTGGTGACGGTGGTTTCGGTAACCGTAACCGCAATAACCAAGGTGGTAATCAAGGCAACTTCCGTGGTCA
AGGAAGAATGAACATGAACCAGAATCAGAACCGTGGCAACAATGCCAACAATTTCGGTGGCAACACAAATGCCGGTGGTAACA
ACTTCCAAGCTTCCCCCGGAAATCAAAACCGTGGTGGACAAAACATGAATCGTGGTAATCAACAGAACCAGAACCGTGCTGGT
GGTAACATGAACCAAGGCAACAACATGAATCAGGCCGGCGGCGGAAATGTTAATCGCCCTCAGCAAAATAAGCCAATGAACAT
GAATACCGGTAACCAAAATCAAAATAGAAACAACCAGGTTGGTGGCGGCGGCAATATGAACCGCAACCAAAACCAAAATAACC
GCAACAACTTCGACAATGACAATTCCTTTGGCGGAGGAAACAACTTTGGCAACAACCGCAACAACAATTTTTCCAATGACAGT
TTTGGTAGCAACGATAATAACTTTGGTGGTAACAATAGTGGTAGTAACAACTTCTCGAATGACAACAACTTCGGTGGTAATAA
CCGTGGTAACAACTTCGGTGGTAACAAGCGCAACCAGAACGATAACTTTAATAACTCGTTCGGTAGCGGAAACAACAGCGGTG
GAAACAATCGCTTCAATAACGATGGAAACAATTTCAATAGCAATTTTAACAACCGCAACCAAAACAATCGTTTTGACAACGAC
AACGATAACAATTTTACTGGTAATAAGGGTGGTAACAATTTCGGCAACCGTCAAAACAAGCCCTTCAATCAGGGAAACAATCA

GGGTGGCAACCAGGGAGGCGGTATGTCCTTTGGTGAATTTAACAACTCCAATTCCTCCAACCGTAGTGGTGGAAACTTCGGTG
GAAACAATTCCGGTTTCAATTCTGGCAACGCCGGAGGAAACAAGTGGAACAACAATTCCCAGCAATCCAATACTGGCAGCCGC
CTCGCTGGTAGCATTTTTAGCCGACGCTTCTAA
