# Supplementary material for: aBravo Is a Novel Aedes aegypti Antiviral Protein That Interacts with, but Acts Independently of, the Exogenous siRNA Pathway Effector Dicer 2
Source: Viruses. 2020 Jul 11;12(7):748. doi: 10.3390/v12070748 (PMC7411624; doi:10.3390/v12070748)
Supplement: Supplementary file 1 [file viruses-12-00748-s001.zip › Supplementary File S2.pdf]

Supplementary File S1. Multiple sequence alignment of Q0IFK9 homologs in other mosquito species. Multiple sequence alignment using Clustal Omega method was conducted in Geneious Prime v.2020.1.2.

```
Q0IFK9_Ae._aegypti                      MSETEKKAE-QS--------TGGETETDGPKPDAEHQRKINVANLPWDVTEQQLRDLFSG
KXJ69968_Ae._albopictus                 MTETDKKAE-QS--------TG-A-ETEGPKPDAEHQRKINVANLPWDVTEQQLRDLFSG
XP_019549434_Ae._albopictus             MSQVEKKTE-QS--------TG-EHETDTSKGNSEYQRRINVANLPWDVTEQQLRNLFSD
XP_563491_A._gambiae                    MTDAEKTAPETTAETKETTKAENDGTEGDVQKDNEHQRTINVGNLPKDITEQQLRDHFAG
XP_001845014_C._quinquefasciatus        MSDATKPVEKTQ--------DGTENEGDAPKTDNEHQRKINVGNLPSDITEQQLRDHFSG
XP_001845020_C._quinquefasciatus        --------------------------------MCDNGKKLNVGNLPVDVTEKELRDHFKE
XP_312269_A._gambiae                    ------------------------MADNPSRMKADNKKKLNVGNLPADITEEELRELFAD
XP_001662345_Ae._aegypti                ----------------------MATAENTVNKAADNGKKLNVGNLPADVTEDELREHFKD
XP_019563176_Ae._albopictus             ----------------------MASAENTVNKAADNGKKLNVGNLPNDVTEDELREHFKD
KFB46015_A._sinensis                    ------------------------MAENPSTMKADNKKKLNVGNLPADTTEEELREHFAD
KFB46017_A._sinensis                    MTETEKNVVTTKTEADES--MNVDKEGNDVQKDNEHQRTINVGNLPKDVTEQQLREHFAG
ETN59818_A._darlingi                    ------------------------MAENPSTMKADNKKKLNVGNLPNDVTEEELREHFAD
XP_003436203_A._gambiae                 MTDAEKTAPETTAETKETTKAENDGTEGDVQKDNEHQRTINVGNLPKDITEQQLRDHFAG
                                                                          :  : :**.*** * **.:**: *

Q0IFK9_Ae._aegypti                      HEVEKVEIYHYRKYTLALVLFKDKDTAAAALAEKENSMFRDRRLRMHMEFTSIRHIKREV
KXJ69968_Ae._albopictus                 HEVEKVEIYHYRKYTLALVLFKDKDTAAAALADKENAMFRDRRLRMHMEFTTIRHIKREV
XP_019549434_Ae._albopictus             HEVEKVDIYHYRRYTVAVLFKNKETKAAALAGKENFMFGDRRLRMYSELTYIRYIKREL
XP_563491_A._gambiae                    HEVERVEIYHYLRNTLALLVFKEKAAAQKACEEKDGSMLNDRRLRIHIEYITIRYTKKDV
XP_001845014_C._quinquefasciatus        YEIERVDIYHYRKYTLALLLFKEKDAAAKAVEEKEHSTLRGRRIRVHLEYMTIRHIKREV
XP_001845020_C._quinquefasciatus        FTVENVEIFHYLKYTLALLLFKDKDTATKALKAKEGTLFRNRRLRMHIEYIAIWNIKKNV
XP_312269_A._gambiae                    HPVESVEIFHYKQYTLALLLFKDKETATKALKEKENSVFRGRRLRMHLEFIVICHMKKDV
XP_001662345_Ae._aegypti                FTVENVEIFHYLKYTLALLLFKDKDTATKALKEKEGSMFRNRRLRMHIEYIAIWNIKKNV
XP_019563176_Ae._albopictus             FTVENVEIFHYLKYTLALLLFKDKDTATKALKEKEGSMFRNRRLRMHIEYIAIWNIKKNV
KFB46015_A._sinensis                    HPVESVEIFHYKQYTLALLLFKDKETATKALKEHDNSVFRGRRLRMHLEYIVICHMKKDV
KFB46017_A._sinensis                    HEVERVEIYHYHRNTLALLLFKDKVAAQKAIDEKDGKMMGDRRLRIHLEYITIRYTKKDV
ETN59818_A._darlingi                    HPVESVEIFHFKQYTLALLLFKDKETATKALKEKENSVFRGRRLRMHLEYIVICHMKKDA
XP_003436203_A._gambiae                 HEVERVEIYHYLRNTLALLVFKEKAAAQKACEEKDGSMLNDRRLRIHIEYITIRYTKKDV
                                        . :* *:*:*: : *:* ::**:* :   *   ::   : .**:*:: *   *   *::

Q0IFK9_Ae._aegypti                      IVTVVEDENMTEEQLWDKVKAAGVEPTSVLIFYPLGYVTLEKECDKAPILEKLKAAGFNA
KXJ69968_Ae._albopictus                 VVTVIEDENMTEEQLWDKVKAAGVEPTSVLIFHPLGYVTLEKECDKPALLEKLKTAGFNV
XP_019549434_Ae._albopictus             VVTAIEDENMTEEQLWDKVKAAGVEPTSVLIFYPLGYVTLEKDCDKPAVLEKLKAAGFNA
XP_563491_A._gambiae                    IVAVLDD-DMTEEKLYDRVKE-HCEVTQVFIFHPLGYVHLGKDVDKAAVLEKLNAGGLKA
XP_001845014_C._quinquefasciatus        IVTVLDDENTTEDQLWDKIKAAGVEVTSVLVFYPLGYVALDKEADKPAILEKLTAAGFNA
XP_001845020_C._quinquefasciatus        FVYVVDD-NTTEEDVYDKFKT-ITEVTGVLVFHPLAYVSCNTQEQKEAAIKELNAEETTV
XP_312269_A._gambiae                    FVHVVDNENVTEEDVYEKFKD-LAKVQSVVLFHPLAYVTCTSPEDKEEAMKKIVEAGINV
XP_001662345_Ae._aegypti                FVTVVDD-NTTEEEVYDKYKD-VTEITGVLVFHPLAYVSCKTQEQKEAAIKELNSEEVKV
XP_019563176_Ae._albopictus             FVTVVDD-NTTEEQVYDKYKD-VTEITGVLVFHPLAYVSCKTQEQKETAMKELNSEEVTV
KFB46015_A._sinensis                    FVYVVDNENVTEEDVYEKFKE-LAKLQSVVLFHPLAYVTCTSPEDKEEAMKKIAEAGINV
KFB46017_A._sinensis                    IVTVVDD-DMTEEKLYDRVKE-HCEVTQVFIFHPLGYVHLGKEADKTVVLEKLNASGLKA
ETN59818_A._darlingi                    FVYVVDNENVTEEDVYEKFKD-LAKVQSVVLFHPLAYVTCTSPEDKEEATKKIAEAGINV
XP_003436203_A._gambiae                 IVAVLDD-DMTEEKLYDRVKE-HCEVTQVFIFHPLGYVHLGKDVDKAAVLEKLNAGGLKA
                                        .* .::: : **:.:::: *    :   *.:*:**.**    .  :*    :::     ..

Q0IFK9_Ae._aegypti                      HDLNQRDQNQHLDIWRAAKLFFRNRNRVQLLNIPNKWIEDKEEMKKQCAEGGTITEAVSN
KXJ69968_Ae._albopictus                 HDVNQRDQNQHLDIWRAAKLFFRNRNRVQLLNIPNKWIEDKEEMKKQCAEGGTITEAVSN
XP_019549434_Ae._albopictus             HDVNQRDQNHHLDIWREATLFFRNRNRVQLLNIPSKWIEDKEEMKKQCAEGGTIIEAVTS
XP_563491_A._gambiae                    YDVNGRDQNQHVDLWRAAKLFFRNRNRVQLLNIPQKWVENTEELKKACSEAGTITEAIAN
XP_001845014_C._quinquefasciatus        HDVNQRDQNQHLDIWRAAKLFFRNRNRVQLLNIPTKWIEDKEEMKKQCAEGGVITEAVSN
XP_001845020_C._quinquefasciatus        YDLTGHDQNHHIEIWRAAKLFFRNMNRVSLINLPESWVSNQEELKKAVEGTGTLTEVKVI
XP_312269_A._gambiae                    YDCNGHDQNQHIDVLRAAKLFFRNLNRVQMYNIPESWISSQDELKKAVESSGTVTEVRVT
XP_001662345_Ae._aegypti                YDVTGHDQNHHIEIWRAAKLFFRNMNRVSLINLPESWVSNQEELKKAVAHTGTTTEVKVI
XP_019563176_Ae._albopictus             YDVTGHDQNHHIEIWRAAKLFFRNMNRVSLINLPESWVSNQEELKKAVAHTGTTTEVKVI
KFB46015_A._sinensis                    YECHGHDQNQHIDVLRAAKLFFRNLNRVQMYNIPENWVNSQDELKKAVESSGTVTEVRVT
KFB46017_A._sinensis                    YDVNGRDQNQHVDLWRAAKLFFRNRNRVQLLNVPQKWVENTDELKKACSEAGTITEAVAN
ETN59818_A._darlingi                    YECNGHDQNQHIDVMRAAKLFFRNLNRVQMYNIPESWVNSQDELKKAVEGSGTVTEVRVT
XP_003436203_A._gambiae                 YDVNGRDQNQHVDLWRAAKLFFRNRNRVQLLNIPQKWVENTEELKKACSEAGTITEAIAN
                                        ::   :***:*::: * *.***** ***.: *:* .*:.. :*:**     *.  *.

Q0IFK9_Ae._aegypti                      NVTQNPQMASNYARIFYETEEQAVKAAKALNGRVVDGKRIHALHLGSALMPNYKTSVYVT
KXJ69968_Ae._albopictus                 NVTQNPVVASNYARIFYETEEQAAKAAKALNGRVVEGKRIHALHLGSALMPNYKTSVYVT
XP_019549434_Ae._albopictus             NAAQNPSVIPNYARIFYETEEQAAKAAKALNGRVVEGKRIHALYPGIVLMPNYKTSVYVT
XP_563491_A._gambiae                    NVSQGS--SNYYARIFFETEEQAAKAAELLNGKVFEGKRIHALHLSSALLPNYKTSVYVA
XP_001845014_C._quinquefasciatus        NVNQS---SSNYARVFYETEEQAIKAAASLNGRIVEGKRIHALHLGSALIPNYKTSVYVT
XP_001845020_C._quinquefasciatus        NNSHGS-----VAQLFYEDEDTARNASQTLNQQVFEGKRIHALHVTAALIPDYTSSVYLT
```

```
XP_312269_A._gambiae                  TGNFGP-----AAQVFYETADEAKAAAEKLNQQLFEGKRVHAHHVIATMIPNFKTSVYVE
XP_001662345_Ae._aegypti              NNSHGA-----VAQLFYENEETARKAALELNQKIFDGKRIHALHVTAALIPNYETSVYLT
XP_019563176_Ae._albopictus           NNSHGA-----VAQLFYENEETAKKAALELNQKIFDGKRIHALHVTAALIPNYETSVYLT
KFB46015_A._sinensis                  TGNFGP-----AAQVFYESAEEAKAAADKLNQQIFEGKRIHAHHVIATMIPNYKTSVYVE
KFB46017_A._sinensis                  NVNQAA--SNYYARIFFETEEQAAKAAELLNGKVFEGKRIHALHLSSALLPNYKTSVYAS
ETN59818_A._darlingi                  TGNFGP-----AAQVFYETADEAKAAADKLNQQLFEGRRIHAHHVIATMIPNYRTSVYIE
XP_003436203_A._gambiae               NVSQGS--SNYYARIFFETEEQAAKAAELLNGKVFEGKRIHALHLSSALLPNYKTSVYVA
                                      .           *::*:*  : *  *:  ** ::.:*:*:** :   .::*:: :***


Q0IFK9_Ae._aegypti                    SLDKGVTEEQVYDHFKQFGEIEFTNRRNCGD-AAIICYKNAASAEKALACTTFPAPTEEN
KXJ69968_Ae._albopictus               ALDRGVTEEQVYDHFKQFGEIEFTNRRNCGD-AAIICFKNAASAEKALACTTFPAPTEEN
XP_019549434_Ae._albopictus           SLSKGVTEEQVYDYFKQFGEIEFTSRRNCGD-AAIICFKNAASVDKALACTTFPAPTEEN
XP_563491_A._gambiae                  PLERTVTEEEVYEHFKQFGDIDFVNRRNCGE-NAIVCYKTSEAAEKALACTTFPAPTEAN
XP_001845014_C._quinquefasciatus      SLEKTVTEENVYDHFKQFGEIEFVNRRNCND-AAIVCFKSADSVEKAIACTKLPAPTEAD
XP_001845020_C._quinquefasciatus      GLEKPISEETIYSHFEQFGEIEFVSRRKCADEHAIICFKNSSAVEAALECKTLPVPKPDS
XP_312269_A._gambiae                  NLDKTITEEMLYAHFEQYGEIDFVNRRKFGDEHGMVCFKEASSVDRALECTTLPVTKKEG
XP_001662345_Ae._aegypti              SLEKATSEETIYDHFKQFGEIEFVSRRKCADEHAVICFKNASAVEKALECKTLPITKPLS
XP_019563176_Ae._albopictus           SLEKATSEETIYDHFKQFGDIEFVSRRKCADEHAVICFKDASAVEKALECKTLPVPKADS
KFB46015_A._sinensis                  SLDKSVTEEILYDHFQQYGEIDFVNRRKFGDEHGLVCFKEASSVERALECTALPMPKKED
KFB46017_A._sinensis                  PLEKTVTEEDVYDYFKQFGEIDFVNRRNCVD-NAIVCFKSEDAVEKALACTTFPAPTEAN
ETN59818_A._darlingi                  QLDKSITEEQLYDHFLQYGEIEFVNRRKFGDEHGLVCFKEESSVEKALLCTALPLPKKEV
XP_003436203_A._gambiae               PLERTVTEEEVYEHFKQFGDIDFVNRRNCGE-NAIVCYKTSEAAEKALACTTFPAPTEAN
                                       *.:  :** :* :* *:*:*:*..**:  :  .::*:*   :.: *: *. :* ..

Q0IFK9_Ae._aegypti                    K---------------------------------DATKEVVVKKYDGPLVLRPVALKRKL
KXJ69968_Ae._albopictus               K---------------------------------DATKEVAVKKYDGPLVLRPVALKRKL
XP_019549434_Ae._albopictus           K---------------------------------DATKEVVVKKYDGPLVLRPFTFKRKL
XP_563491_A._gambiae                  K---------------------------------EATKTVTVLRYDGPLVLRATSVKRKA
XP_001845014_C._quinquefasciatus      K---------------------------------DATTEITVKKYDGPLLLRTLAMKRKL
XP_001845020_C._quinquefasciatus      EE--------------------------------TEDKTIGVKKYNGPLIIDLKPSHVKK
XP_312269_A._gambiae                  EE------------------------------TTTEDKTIVVKRYDGPLVLDIKLPAMKK
XP_001662345_Ae._aegypti              DV--------------------------------TEERAITVKRYNGPLIIDLKPSHVKK
XP_019563176_Ae._albopictus           DK--------------------------------TEDRAITVKKYNGPLIIDLKPSHVKK
KFB46015_A._sinensis                  E-------------------------------TANVEKTIVVKRYDGPLVLDIKLPPIKK
KFB46017_A._sinensis                  K---------------------------------EATKTITVKRYDGPLVLRAAGVKRKQ
ETN59818_A._darlingi                  GSEEEAAPAATTTTDGTASATAAATTSAAAVTPTETEKTIVVKRYDGPLVLDIKLPQLKK
XP_003436203_A._gambiae               K---------------------------------EATKTVTVLRYDGPLVLRATSVKRKA
                                                                             : * :*:***::       *

Q0IFK9_Ae._aegypti                    KAGEEE---------------KDQAEKTKEQQSVLGKLKAYHPIFVGNVPLNCPPLELKH
KXJ69968_Ae._albopictus               KPGEEE---------------KDQAEKTKEQQSVLNKLKAYHPIYVGNVPLNCPPLELKH
XP_019549434_Ae._albopictus           ESTEGE---------------KVKTEKTKEQRSVLHKLKAYHPIYVGNVPFNCPPLELKQ
XP_563491_A._gambiae                  ASGTAKGAAGEGKTRESRP--AGGSDREKSHKDILQKLQAFYPVYVSNIPFSCPAHVIRE
XP_001845014_C._quinquefasciatus      KTNEDGTP-------------ADPEEKTKEMADVLKKLQAYHPVFVGNVPLNCPPLELKR
XP_001845020_C._quinquefasciatus      --LLED-------------------GEESKMRPPPLPLAKLWPIYVSNLPYTADKRDMRD
XP_312269_A._gambiae                  GASSEE-------------------GEEQKQRPPPQPLAKLWPIYVANLPYKADKREIKQ
XP_001662345_Ae._aegypti              --LLED-------------------GEESKMRPPPLPLAKLWPIYVSNLPYTADKRDIRD
XP_019563176_Ae._albopictus           --LLED-------------------GEESKMRPPPLPLAKLWPIYVSNLPYTADKRDIRD
KFB46015_A._sinensis                  NSTGEE-------------------GEEQKQRPPPQPLAKLWPIYVANLPYKADKREIKQ
KFB46017_A._sinensis                  AATTK-RTAPDGKTREPRTIGAAGAERERTHKDILQKLQAFWPVYVSNVPYSCPSHVIRD
ETN59818_A._darlingi                  GATSEE-------------------PEEQKQRPPPAPLAKLWPIYVANLPYKADKREIKK
XP_003436203_A._gambiae               ASGTAKGAAGEGKTRESRP--AGGSDREKSHKDILQKLQAFYPVYVSNIPFSCPAHVIRE
                                                                .          *    *::*.*:* ..    ::

Q0IFK9_Ae._aegypti                    YFA-QHGPIKFMFSPQI-LAYRNSAPHPVKTFIIYYMNRPSQQNAVRFFDHKFFGGHRLH
KXJ69968_Ae._albopictus               YFA-QHGPIKFMFSPQI-LSYRNSAPHPVKTFIIYYMNRVSQQNAVRFFDHKFFGGHRLH
XP_019549434_Ae._albopictus           YFA-QHGPIKFMFSPQI-FSYRNSAPHPVKTFIVYYMNRESQQNAVRFFDHKFFGGHRLH
XP_563491_A._gambiae                  FFSGAGGEVKFIFSPQHYHPYRLSSPQPVKTAMVYYTRRSDMLNAIKQLDKKMLNNQHLH
XP_001845014_C._quinquefasciatus      YFA-QHGPIKFMFSPQI-VTYQTSAPHPVKTFIIYYNFRPSALNACKFLDHKFFGGHRLH
XP_001845020_C._quinquefasciatus      YFSSVGGHIKFIFSPNI-PAYKTSQTSMVMAALIYYARREQATDAIKAFNGKHFQNRCLH
XP_312269_A._gambiae                  YFSSYGGHIKFIFSPNI-PSYKQSQTSMVMAALIYFARREQANEAIKAFNGKHFQSKCLH
XP_001662345_Ae._aegypti              YFSSVGGHIKFIFSPNI-PAYKTSQTSMVMAALIYYARREQATDAIKAFNGKHFQNRCLH
XP_019563176_Ae._albopictus           YFSSVGGHIKFIFSPNI-PAYKTSQTSMVMAALIYYARREQATDAIKAFNGKHFQNRCLH
KFB46015_A._sinensis                  YFSSYGGHIKFIFSPNI-PSYKQSQTSMVMAALIYFARREQANEAIKAFNGKHFQSKCLH
KFB46017_A._sinensis                  LFSSQGGEVKFLFSPQQYLTYRLSAPQPVKTVMLYYTRRNDMYNALKHFDKKALLGHHLN
ETN59818_A._darlingi                  YFSSFGGHIKFIFSPNI-PSYKQSQTSMVMAALIYFARREQANEAIKAFNGKHFQSKCLH
XP_003436203_A._gambiae               FFSGAGGEVKFIFSPQHYHPYRLSSPQPVKTAMVYYTRRSDMLNAIKQLDKKMLNNQHLH
                                       *:   * :**:***:   .*: * .  * : ::*:  * .  :* : :: * : .: *:
```

```
Q0IFK9_Ae._aegypti                    VLPLRGTQNMDVKKTIKMTKIP-FMSEDALFKKIKPFVGKINRIVKKSRLIAYIELEDSA
KXJ69968_Ae._albopictus               VLPLRGTQNMDVKKTIKMTKIP-FMSEDALFKKIKPFVGKINRIVKKARLAAYIELEDTA
XP_019549434_Ae._albopictus           VLPLRGAQCMDVKKTIKMTQLP-SMSEDALFKKIKPFVGKINRIVKKVRFEAFIELEDTA
XP_563491_A._gambiae                  VLPGRGDCNFSQQKTVKLSKITESLSEDAIFRKMRP-LGKVVRLTKKNRSLAFVEFADAA
XP_001845014_C._quinquefasciatus      VLPLRGELNFDVKKTVKITKIP-YLSEDALFKKVKPYVGKINRIVKKTRVAAYIELNDLA
XP_001845020_C._quinquefasciatus      VFPGRKDTYFNPETSVRLVRLTIGVSEEKLFEKFRK-FGFIECVVKRNRNTALIEFRDKE
XP_312269_A._gambiae                  VFPGRKDTYFDVEKSIKVVRLNLAVTEEKLFEKFRK-FGFIECVVKKDRTTAYIEFRDKE
XP_001662345_Ae._aegypti              VFPGRKDTYFNPETSVRLVRLTIGVTEEKLFEKFRK-FGFIECVVKRNRNTALIEFRDKE
XP_019563176_Ae._albopictus           VFPGRKDTYFNPETSVRLVRLTIGVTEEKLFEKFRK-FGFIECVVKRNRNTALIEFRDKE
KFB46015_A._sinensis                  VFPGRKDTYFDTEKSIKVVRLNLAVTEEKLFEKFRK-FGFIECVVKKDRTTAFIEFRDKE
KFB46017_A._sinensis                  VIPGRGESNFEQQNTVRLSRINESLSEDVIFRKMCP-LGKIVRLTKKSRSLAFVEFADAA
ETN59818_A._darlingi                  VFPGRKDTYFDTEKSIKVIRLNIAVTEEKLFEKFRK-FGFIECVVKKDRTTAYIEFRDKE
XP_003436203_A._gambiae               VLPGRGDCNFSQQKTVKLSKITESLSEDAIFRKMRP-LGKVVRLTKKNRSLAFVEFADAA
                                      *:* *    :. :.:::: ::   ::*: :*.*.   .* :  :.*: *  * :*: *


Q0IFK9_Ae._aegypti                    DVEKMMNVETPDHPLPKGKMEQLKTPISARLYNEDDVRVLNGIAKIVAKNPDMLKKNIQ-
KXJ69968_Ae._albopictus               DVDKMMNTETADHPLPKGKMEQLKTPISTRVYNEDDVRVLNGIAKIVAKNPDMLKKTPLP
XP_019549434_Ae._albopictus           DVVKLMKTETPNHPLPKSKMEQLTTPISVRLYDEDDVRILNGIAKIVEKNPDMLKKTVLP
XP_563491_A._gambiae                  DVEKVLKMKPDELTINC-VYSKITKDVSRRLYNESDPRILGTIVRLLRRNPKMLNKTTGA
XP_001845014_C._quinquefasciatus      DVDKFMDVDAADHPLNKAKMEPISANTSVRLYDENDTRVLRGIAKIIAKNPDMLKKVAPG
XP_001845020_C._quinquefasciatus      VCDKVLQLPDGEKPVRC-GIEPLTTKVNRKIFKENDEKISLAMQEIIDKNPAVLENVQSN
XP_312269_A._gambiae                  IAEKVLKLDEKQRPVRC-NVEAVTGKINKKIFKENDEKISLAMQEIIDKNPAVLENVSSD
XP_001662345_Ae._aegypti              VCDKVLQLPDSEKPVRC-GIEPLTTKVNRKIFKENDEKISLAMQEIIDKNPAVLENVQST
XP_019563176_Ae._albopictus           VCDKVLQLSDSEKPVRC-GIEPLTTKVNRKIFKENDEKISLAMQDIIDKNPAVLENVQST
KFB46015_A._sinensis                  IAEKVLKLDEKQRPVRC-NVEPVTGKINKKIFKENDEKISLAMQEIIDKNPAVLENVSSD
KFB46017_A._sinensis                  DVEKILKMKQEELPINC-VYSKITKDVNRRLYNESDPRIVGTIVRLMRRNPKMLTKTTGA
ETN59818_A._darlingi                  IAEKVLKLDEKQRPVRC-LVEPVTGKINKKIFKENDEKISSAMQEIIDKNPAVLENVSSD
XP_003436203_A._gambiae               DVEKVLKMKPDELTINC-VYSKITKDVSRRLYNESDPRILGTIVRLLRRNPKMLNKTTGA
                                         *.:.    : .:     . :.   . :::.*.* ::   :  :: :** :* :

Q0IFK9_Ae._aegypti                    -PP------------------NKRPRMEFGGPGFNQGPG----------GFVPNN-----
KXJ69968_Ae._albopictus               -PP------------------NKRPRMDFGGPGFDQGPG----------GFNP-------
XP_019549434_Ae._albopictus           --L------------------NKRPRMDFGGPGFNQGPG----------GFNP-------
XP_563491_A._gambiae                  GPLMGGN--TAVGGGPGGFGGGKRPRMNGPPGGKLFFLIDLLVCFTLHRLYFPVHFHNVE
XP_001845014_C._quinquefasciatus      -QL------------------NKRPRLN-GPGTFNQGPV----------GFNPSN-----
XP_001845020_C._quinquefasciatus      RF--SGP------------PPLKRGRFNGPDPNFGNLGNR--------NDFNSMN-----
XP_312269_A._gambiae                  RS--SGP------------PPLKRSRFSGPA-NFGMQGPR--------NQFN-PN-----
XP_001662345_Ae._aegypti              RF--SGP------------PPLKRGRFNGPDPNFGG--NR--------GD---FN-----
XP_019563176_Ae._albopictus           RF--SGP------------PPLKRGRFNGPDPNFG---NR--------GD---FN-----
KFB46015_A._sinensis                  RS--SGP------------PPLKRSRFSGPS-NFGMQGPR--------SQFN-PN-----
KFB46017_A._sinensis                  GPLMSRGPMNVGGGGPGRFAGGKRPRMSGPPGFGNP-PP-----------FNPNNGGNLN
ETN59818_A._darlingi                  RS--SGP------------PPLKRGRFSGPA-NFGMQGPR--------NQFN-AN-----
XP_003436203_A._gambiae               GPLMGGN--TAVGGGPGGFGGGKRPRMNGPPGGFGNVPP-----------FNPNAGG---
                                                            ** *:.

Q0IFK9_Ae._aegypti                    ------------------NMNNPNAIQDLLRLAFMSGKNVGESLASNQS-----------
KXJ69968_Ae._albopictus               ------------------NMNNPNAIQDLLRLAFMSGKNVGENLASNQS-----------
XP_019549434_Ae._albopictus           ------------------NMNNPNAIQDLLRLAFLSGKNVGESLASNQS-----------
XP_563491_A._gambiae                  MHFCLLNSSTHSAFARRNSSISRYKFAYFLLLFFHSNFH---------------------
XP_001845014_C._quinquefasciatus      ------------------NMNNPQAIQDLLRLAFMSGKNVGESLATGQG-----------
XP_001845020_C._quinquefasciatus      ---------------SNNDFSNPQVIQDLLRLAFISGKNLGEGLASGNN-----------
XP_312269_A._gambiae                  ---------------NGGDFSNPQVIQDLLRLAFISGKNLGEGLAISGN-----------
XP_001662345_Ae._aegypti              ---------------NGNDFSNPQVIQDLLRLAFISGKNLGEGIASGNN-----------
XP_019563176_Ae._albopictus           ---------------NGNDFSNPQVIQDLLRLAFISGKNLGEGIASGNN-----------
KFB46015_A._sinensis                  ---------------AGNDFSNPQVLQDLLRLAFISGKNLGEGLANGGQ-----------
KFB46017_A._sinensis                  ---------------------NNQAIQDLLRLAFMSGKNVGETLAAGGG--SGNQGPNRG
ETN59818_A._darlingi                  ---------------SATDFSNPQVIQDLLRLAFISGKNLGEGLAISGN-----------
XP_003436203_A._gambiae               ------------NNLNNNQA-----IQDLLRLAFMSGKNVGESLAAGGGNVGGNQGPNRG
                                                               :  :* * * *. :

Q0IFK9_Ae._aegypti                    -----SF--GPDPPVANSNN------FGDGGFGNRNR------NNQ-GGNQGNFRGQGRM
KXJ69968_Ae._albopictus               -----SF--GPDPPVANSNN------FGDGGFGNRNR------NNQ-GGNQGNFRGQGRM
XP_019549434_Ae._albopictus           -----SF--GSDSSIANSTN------FGDGSFGNRNR------NSQ-GGNQGNIRGQGKM
XP_563491_A._gambiae                  ------------------------------------------------------------
XP_001845014_C._quinquefasciatus      -----SFN-APDPPLANSNN------FGDGGFRGNNNRGGN--NQM-GGGQSNFRPNNRN
XP_001845020_C._quinquefasciatus      ----NPFNNSSDSPLSSLNLANG--------FSGRNFGGGSNNGFEQ-------------
XP_312269_A._gambiae                  ---SNPFN-SGDPPLPNLN-L-T--------N-GFGG-GNQRNSF-NNSNNGNFGGS---
```

```
XP_001662345_Ae._aegypti                 ----NPFN-SSDSPLSGLNLSNG--------FSGRNFGGGSNNFGNNAGNRNNFGGN---
XP_019563176_Ae._albopictus              ----NPFN-SSDSPLSGLNLSNG--------FSGRSFGGGSNSFGNNPGNRGNFGGN---
KFB46015_A._sinensis                     -HGNNPFT-GGDPPLPSLNNL-G--------N-GFGGGGGQRNNF-NNSNNGNFGGS---
KFB46017_A._sinensis                     GGGGNSFN-NADPPISNQGGGNNAFGGGDGGFGAGGGGGGGGRNMNNRNQN-NFRGGNRN
ETN59818_A._darlingi                     ---SNPFN-SGDPPLANLNNG----------F-GGGSGGGNRNNF-NNSNSGNFGGN---
XP_003436203_A._gambiae                  GGGGGSFN-NADPPISNQGGGNNAFGGGDGGFGAGGGGGGGGRNMNNRNQNMNFRGGNRN

Q0IFK9_Ae._aegypti                       NMNQNQNRGNN--ANN--FGGNTNAGGNNFQASPGNQN-RGGQNMNRGNQQ-NQNRAG--
KXJ69968_Ae._albopictus                  N--QNQNRGNN--AGN--FGGNNNAGGNNFQGSPGNQN-RGGQNMNRGNQQ-NQNR-G--
XP_019549434_Ae._albopictus              N--QN-------------SGGNNNAGGNNFQGSPGNQN-RGGQNMNRVNQQ-NQNR-S--
XP_563491_A._gambiae                     ------------------------------------------------------------
XP_001845014_C._quinquefasciatus         NMNNQNNQNRP--N-----NNFNNAGGNNFQGNAQNAN-RGNNQGNANNRNPSQNRVQGQ
XP_001845020_C._quinquefasciatus         ------------------------------------------------------------
XP_312269_A._gambiae                     ---------------------SNNASGNMGGGNR-NNM--GGGGVNRNFG-QNVGGGGGA
XP_001662345_Ae._aegypti                 ---------------------PNNPGQRNFGGQ---------Q-RNPNNPGNAFGGGNP-
XP_019563176_Ae._albopictus              ---------------------LNNPGQRNFGGN---------QQQNRNNPGNAFGGGSNQ
KFB46015_A._sinensis                     ---------------------SNNAGGNMGGN-R-NNM---GGGVNRNFG-QNVGGGGGG
KFB46017_A._sinensis                     NANNNVGGNRGLGGGNLGNSNNNNAGGNNNFGNNQNQG-VGGGGGPRSGGGALGG-GNSG
ETN59818_A._darlingi                     ---------------------SNNSGGGGNMGNRGGNMGGGSGGLNRNFGGQNLGGGGNG
XP_003436203_A._gambiae                  NQNNNVGGNRGLG-----NNSNNNAGGNNNFGNQN-QS-GGGSGGPRGGGGALGG-GGGN


Q0IFK9_Ae._aegypti                       ----GNMNQ----------------GNNMNQAGGGNVN--RPQQNKPMNMNT--------
KXJ69968_Ae._albopictus                  ----GNMNQ----------------GNNMNQS-GGNVN--RPQQNKPMNMNT--------
XP_019549434_Ae._albopictus              ----GNMNT-----------------SNMNQS-GGNFN--RPQQNKPMNMKT--------
XP_563491_A._gambiae                     ------------------------------------------------------------
XP_001845014_C._quinquefasciatus         GQGQGGVQQNRNVTQQ--------QQNKPNVQGGGNLN--LG-QNRPQNQQG--------
XP_001845020_C._quinquefasciatus         ------------------------------------------------------------
XP_312269_A._gambiae                     MGSGGYGRVNMGG--G---------------FGGKRFNGGSQQTNRMGQ-----------
XP_001662345_Ae._aegypti                 NKFGSSN---------------------------------NQQQNRNLN-----------
XP_019563176_Ae._albopictus              NKFGGGNS--------------------------------NNQQNRNQN-----------
KFB46015_A._sinensis                     GNMGGGYRNNLGG--N---------------FGGKRFNG-SQQSNRMGQ-----------
KFB46017_A._sinensis                     G-GGGGMMNNRNNNPNMNNRNN--NQNMNKRGAGGNVN--VQQQTPMVGGGGIGGN----
ETN59818_A._darlingi                     GGIGGGYRSNLGG--N---------------FGGKRFNG-SQQTNRMGQ-----------
XP_003436203_A._gambiae                  NNSGGGMMNNRNM-NNMNNRNNSNNMNMNNKRTGGNNN--LPQQNAALPAGGVAAAVGGG

Q0IFK9_Ae._aegypti                       ------GN----------------------------QNQNRNNQVGGGGNMNRNQNQNNR
KXJ69968_Ae._albopictus                  ------GN----------------------------QNQNRNNQVGGG-NMNRNQN---N
XP_019549434_Ae._albopictus              ------GN----------------------------Q--NRNNQVGGF-NMNRNQN---N
XP_563491_A._gambiae                     ------------------------------------------------------------
XP_001845014_C._quinquefasciatus         ------GN----------------------------NVNVRNNSQ----TRNQNQGNRNN
XP_001845020_C._quinquefasciatus         ------------------------------------------------------------
XP_312269_A._gambiae                     ----------NNRQ--------GG---------GNQ--NRRF------------------
XP_001662345_Ae._aegypti                 ----------QNR------------------PERNM--NRRF------------------
XP_019563176_Ae._albopictus              ----------QNRQ-----------------GGGNM--NRRF------------------
KFB46015_A._sinensis                     ----------NNRQ--------GG--GGGGGGGGNQ--NRRF------------------
KFB46017_A._sinensis                     ---NKGGLRGNN-QGLGGVGGMGGTVGGGGGGGNNMNMNRSNNNLGGGGGMGGGNNRNNR
ETN59818_A._darlingi                     ----------NNRQ--------GG--GGGG-GGNNQ--NRRF------------------
XP_003436203_A._gambiae                  NMNKGGGMRGNNNQGMG-----GGGSGMGSGGGGNMNMNRSNNSMGGGSGMGGGNNRNNR

Q0IFK9_Ae._aegypti                       NNF-DNDNSFG---GG-NNFGNNRNNNFSNDSFGSND---NNFGGNNS---GSNNFSNDN
KXJ69968_Ae._albopictus                  RNF-DNDNSFG---GG-NNFGNNRNNNFSNDSFGNDD---NNFGNN-----SSNNFSSDN
XP_019549434_Ae._albopictus              SSF-DNDNSFG---GG-NNFGNNRSNNFSNDSFGIDD---NNFDNN-----GSNNFSNDN
XP_563491_A._gambiae                     ------------------------------------------------------------
XP_001845014_C._quinquefasciatus         NNFASDDNSFGSGGGG-GNYNARSNNNFSNNDFSND-----SFGNSND---GGNSFGNQR
XP_001845020_C._quinquefasciatus         ------------------------------------------------------------
XP_312269_A._gambiae                     ------------------------------------------------------------
XP_001662345_Ae._aegypti                 ------------------------------------------------------------
XP_019563176_Ae._albopictus              ------------------------------------------------------------
KFB46015_A._sinensis                     ------------------------------------------------------------
KFB46017_A._sinensis                     NNY-NNDSNS---------SFGGSDSSISSPEYAMITE----------------------
ETN59818_A._darlingi                     ------------------------------------------------------------
XP_003436203_A._gambiae                  NNF-NDTSSFGGVGGGNSNSFGGGNNNFSNDNFGGGRNNDNSYSNDNFGGGRNDSYSSDS
```

```
Q0IFK9_Ae._aegypti                    NFGGN-----NRGNNFGGNKRNQNDNFNNSFGSGNN------SGGNNRFNNDGNNFNSNF
KXJ69968_Ae._albopictus               -FGG------NTNRNFGGNKRNQNDNFN-SFGSGNNS---GSSGGNNRFNNDGNNFSSNF
XP_019549434_Ae._albopictus           -YGG------NTSRNFGGNMRNQNDNFN-SFGSGNNS---GSTGGNNRFINDGNNYKSSF
XP_563491_A._gambiae                  ------------------------------------------------------------
XP_001845014_C._quinquefasciatus      NFNNRQ----NQSDSFGG----------NSFGGGNNRFQNNDNGGNNSFGNNGGNFNN--
XP_001845020_C._quinquefasciatus      ------------------------------------------------------------
XP_312269_A._gambiae                  ------------------------------------------------------------
XP_001662345_Ae._aegypti              ------------------------------------------------------------
XP_019563176_Ae._albopictus           ------------------------------------------------------------
KFB46015_A._sinensis                  ------------------------------------------------------------
KFB46017_A._sinensis                  ------------------------------------------------------------
ETN59818_A._darlingi                  ------------------------------------------------------------
XP_003436203_A._gambiae               NFGGNSGGGGGGRNNFGGNRNN------DSYGSNRNDNFGGSGGGSSNFGG-GN------

Q0IFK9_Ae._aegypti                    NNRNQN-NRFDNDNDNNFTGNKG---GNNFG-----------------------------
KXJ69968_Ae._albopictus               NNRNNQNNRFDND-DNNFSGNTGGNKGGNFG-----------------------------
XP_019549434_Ae._albopictus           NNRSNQNNSFDNG-N-----NFSGNKGGNFG-----------------------------
XP_563491_A._gambiae                  ------------------------------------------------------------
XP_001845014_C._quinquefasciatus      -----RNNRFADNNDN--SDNVGG-NGGGFN-----------------------------
XP_001845020_C._quinquefasciatus      ------------------------------------------------------------
XP_312269_A._gambiae                  ------------------------------------------------------------
XP_001662345_Ae._aegypti              ------------------------------------------------------------
XP_019563176_Ae._albopictus           ------------------------------------------------------------
KFB46015_A._sinensis                  ------------------------------------------------------------
KFB46017_A._sinensis                  ------------------------------------------------------------
ETN59818_A._darlingi                  ------------------------------------------------------------
XP_003436203_A._gambiae               --AFGNNSRFSNDNDGPFSGNSGGNAGGGNNSFGANNRGNNSSNSRFSDDNIGGGGYGGG


Q0IFK9_Ae._aegypti                    --------------------------NRQNKPFNQGNNQGGNQGGGMSFGEFNNSNSSNR
KXJ69968_Ae._albopictus               --------------------------NRQNKPFNQ----GGNQGGGMSFGEFNSNNS--N
XP_019549434_Ae._albopictus           --------------------------NRQNKSFNQ----SGNQGGGMSFGEFNSNNS--N
XP_563491_A._gambiae                  ------------------------------------------------------------
XP_001845014_C._quinquefasciatus      --------------------------NQKNFQRKQFGGNNNQGGGGMSFGEFNSNSNRNN
XP_001845020_C._quinquefasciatus      ------------------------------------------------------------
XP_312269_A._gambiae                  ------------------------------------------------------------
XP_001662345_Ae._aegypti              ------------------------------------------------------------
XP_019563176_Ae._albopictus           ------------------------------------------------------------
KFB46015_A._sinensis                  ------------------------------------------------------------
KFB46017_A._sinensis                  ------------------------------------------------------------
ETN59818_A._darlingi                  ------------------------------------------------------------
XP_003436203_A._gambiae               GNTSNRGGNFNDSGNNFGGNNRGSGGNQKPQGLFNLGGGGGSGGGNL--GGSGNM----S

Q0IFK9_Ae._aegypti                    SGGNFGGNNS------GFNSGNAGGNKWNNNSQQS--------NTGSRLAGSI---FSRR
KXJ69968_Ae._albopictus               RGGNFGGNNS------GFNSGNAGGNKWNNNSQQS--------NTGSRLAAPEGELFVGL
XP_019549434_Ae._albopictus           RSGNFGANNS------GFSPGNAGGNKWNNNSQQS--------NTGSRLAGSI---FSRR
XP_563491_A._gambiae                  ------------------------------------------------------------
XP_001845014_C._quinquefasciatus      FGGN----G----------NGGGGFNKWNNNSNNSGQSNNNQNQSGGRLAG---TIFSRR
XP_001845020_C._quinquefasciatus      ------------------------------------------------------------
XP_312269_A._gambiae                  ------------------------------------------------------------
XP_001662345_Ae._aegypti              ------------------------------------------------------------
XP_019563176_Ae._albopictus           ------------------------------------------------------------
KFB46015_A._sinensis                  ------------------------------------------------------------
KFB46017_A._sinensis                  ------------------------------------------------------------
ETN59818_A._darlingi                  ------------------------------------------------------------
XP_003436203_A._gambiae               YNNNFGGGNSNNSGGNNFRNNDNNKGGWNSNNSGLG--GGRSNNNQDNIGRLAGSLFSRR

Q0IFK9_Ae._aegypti                    F-----------------------------------------------------------
KXJ69968_Ae._albopictus               LLLIVNFLYRVLKPVVRNNRADTFDDERYYQSITPTPALPVPATIQAVAPATAATSVITS
XP_019549434_Ae._albopictus           F-----------------------------------------------------------
XP_563491_A._gambiae                  ------------------------------------------------------------
XP_001845014_C._quinquefasciatus      F-----------------------------------------------------------
XP_001845020_C._quinquefasciatus      ------------------------------------------------------------
XP_312269_A._gambiae                  ------------------------------------------------------------
XP_001662345_Ae._aegypti              ------------------------------------------------------------
```

```
XP_019563176_Ae._albopictus        ------------------------------------------------------------
KFB46015_A._sinensis               ------------------------------------------------------------
KFB46017_A._sinensis               ------------------------------------------------------------
ETN59818_A._darlingi               ------------------------------------------------------------
XP_003436203_A._gambiae            F-----------------------------------------------------------

Q0IFK9_Ae._aegypti                 ------------------------------------------------------------
KXJ69968_Ae._albopictus            DNTFQSEYIPVPRDERYEFPGMMPRDTRMYETGLVSGSDNTISNPVPLYRDERHYYPVST
XP_019549434_Ae._albopictus        ------------------------------------------------------------
XP_563491_A._gambiae               ------------------------------------------------------------
XP_001845014_C._quinquefasciatus   ------------------------------------------------------------
XP_001845020_C._quinquefasciatus   ------------------------------------------------------------
XP_312269_A._gambiae               ------------------------------------------------------------
XP_001662345_Ae._aegypti           ------------------------------------------------------------
XP_019563176_Ae._albopictus        ------------------------------------------------------------
KFB46015_A._sinensis               ------------------------------------------------------------
KFB46017_A._sinensis               ------------------------------------------------------------
ETN59818_A._darlingi               ------------------------------------------------------------
XP_003436203_A._gambiae            ------------------------------------------------------------

Q0IFK9_Ae._aegypti                 ------------------------------------------------------------
KXJ69968_Ae._albopictus            ITSSYILDNRQYEQSINSPSMMHDRLYQPTTHTITSGMLYNDRHYETANSLGSLSNLASA
XP_019549434_Ae._albopictus        ------------------------------------------------------------
XP_563491_A._gambiae               ------------------------------------------------------------
XP_001845014_C._quinquefasciatus   ------------------------------------------------------------
XP_001845020_C._quinquefasciatus   ------------------------------------------------------------
XP_312269_A._gambiae               ------------------------------------------------------------
XP_001662345_Ae._aegypti           ------------------------------------------------------------
XP_019563176_Ae._albopictus        ------------------------------------------------------------
KFB46015_A._sinensis               ------------------------------------------------------------
KFB46017_A._sinensis               ------------------------------------------------------------
ETN59818_A._darlingi               ------------------------------------------------------------
XP_003436203_A._gambiae            ------------------------------------------------------------

Q0IFK9_Ae._aegypti                 ------------------------------------------------------------
KXJ69968_Ae._albopictus            NECHGVEMMMRQDAAAVAAAAVIEHGAAAEVAWGRAEMERQDEIDRQYLVLLPREDKIEP
XP_019549434_Ae._albopictus        ------------------------------------------------------------
XP_563491_A._gambiae               ------------------------------------------------------------
XP_001845014_C._quinquefasciatus   ------------------------------------------------------------
XP_001845020_C._quinquefasciatus   ------------------------------------------------------------
XP_312269_A._gambiae               ------------------------------------------------------------
XP_001662345_Ae._aegypti           ------------------------------------------------------------
XP_019563176_Ae._albopictus        ------------------------------------------------------------
KFB46015_A._sinensis               ------------------------------------------------------------
KFB46017_A._sinensis               ------------------------------------------------------------
ETN59818_A._darlingi               ------------------------------------------------------------
XP_003436203_A._gambiae            ------------------------------------------------------------

Q0IFK9_Ae._aegypti                 ----
KXJ69968_Ae._albopictus            QDEL
XP_019549434_Ae._albopictus        ----
XP_563491_A._gambiae               ----
XP_001845014_C._quinquefasciatus   ----
XP_001845020_C._quinquefasciatus   ----
XP_312269_A._gambiae               ----
XP_001662345_Ae._aegypti           ----
XP_019563176_Ae._albopictus        ----
KFB46015_A._sinensis               ----
KFB46017_A._sinensis               ----
ETN59818_A._darlingi               ----
XP_003436203_A._gambiae            ----
```
