# Supplementary material for: aBravo Is a Novel Aedes aegypti Antiviral Protein That Interacts with, but Acts Independently of, the Exogenous siRNA Pathway Effector Dicer 2
Source: Viruses. 2020 Jul 11;12(7):748. doi: 10.3390/v12070748 (PMC7411624; doi:10.3390/v12070748)
Supplement: Supplementary file 1 [file viruses-12-00748-s001.zip › Supplementary Table S1.docx]

**Supplementary Table S1. Primer and siRNA sequences**

| **Primers sequences for gene knock down** Minimal T7 promoter sequence underlined  Gene specific part **bold** | |
| --- | --- |
| **T7_Q17D57_F** | GTAATACGACTCACTATAGGG **GCTTATCTTCAGGCGAAACTAGG** |
| **T7_Q17D57_R** | GTAATACGACTCACTATAGGG **AAGATACTGTTCCGTTTGCATGT** |
| **T7_Q0IFK9_F** | GTAATACGACTCACTATAGGG **CTATCCATTGGGCTATGTTACTTTG** |
| **T7_Q0IFK9_R** | GTAATACGACTCACTATAGGG **CTGCTCTTCAGTTTCGTAGAAGATT** |
| **dsLacZ_F** | TAATACGACTCACTATAGGG **GTCGCCAGCGGCACCGCGCCTTTC** |
| **dsLacZ_R** | TAATACGACTCACTATAGGG **CCGGTAGCCAGCGCGGATCATCGG** |
| **dseGFP_F** | GTAATACGACTCACTATAGGGG **GCGTGCAGTGCTTCAGCCGC** |
| **dseGFP_R** | GTAATACGACTCACTATAGGGG **TGGTTGTCGGGCAGCAGCAC** |

| **Primers sequences for qPCR** | |
| --- | --- |
| **Q17D57_qL** | TGAACGGGAAGAACTGGACA |
| **Q17D57_qR** | CGTAGTTGTTGTCGTAGCGG |
| **Q0IFK9_qL** | CCAGTCGCCAATTCCAACAA |
| **Q0IFK9_qR** | CCAGTCGCCAATTCCAACAA |
| **S7_L** | CCAGGCTATCCTGGAGTTG |
| **S7_R** | GACGTGCTTGCCGGAGAAC |
| **ZIKV_L** | GTTGTCGCTGCTGAAATGGA |
| **ZIKV_R** | GGGGACTCTGATTGGCTGTA |

| **siRNA sequences** | |
| --- | --- |
| **siQ0IFK9:A** | AGGAAGAGAUGAAGAAACA UU |
| **siQ0IFK9:B** | GGGUGUUACUGAAGAGCAA UU |
| **sieGFP** | ACUUCAAGGAGGACGGCAA UU |
