# Supplementary figures and images for: aBravo Is a Novel Aedes aegypti Antiviral Protein That Interacts with, but Acts Independently of, the Exogenous siRNA Pathway Effector Dicer 2
Source: Viruses. 2020 Jul 11;12(7):748. doi: 10.3390/v12070748 (PMC7411624; doi:10.3390/v12070748)

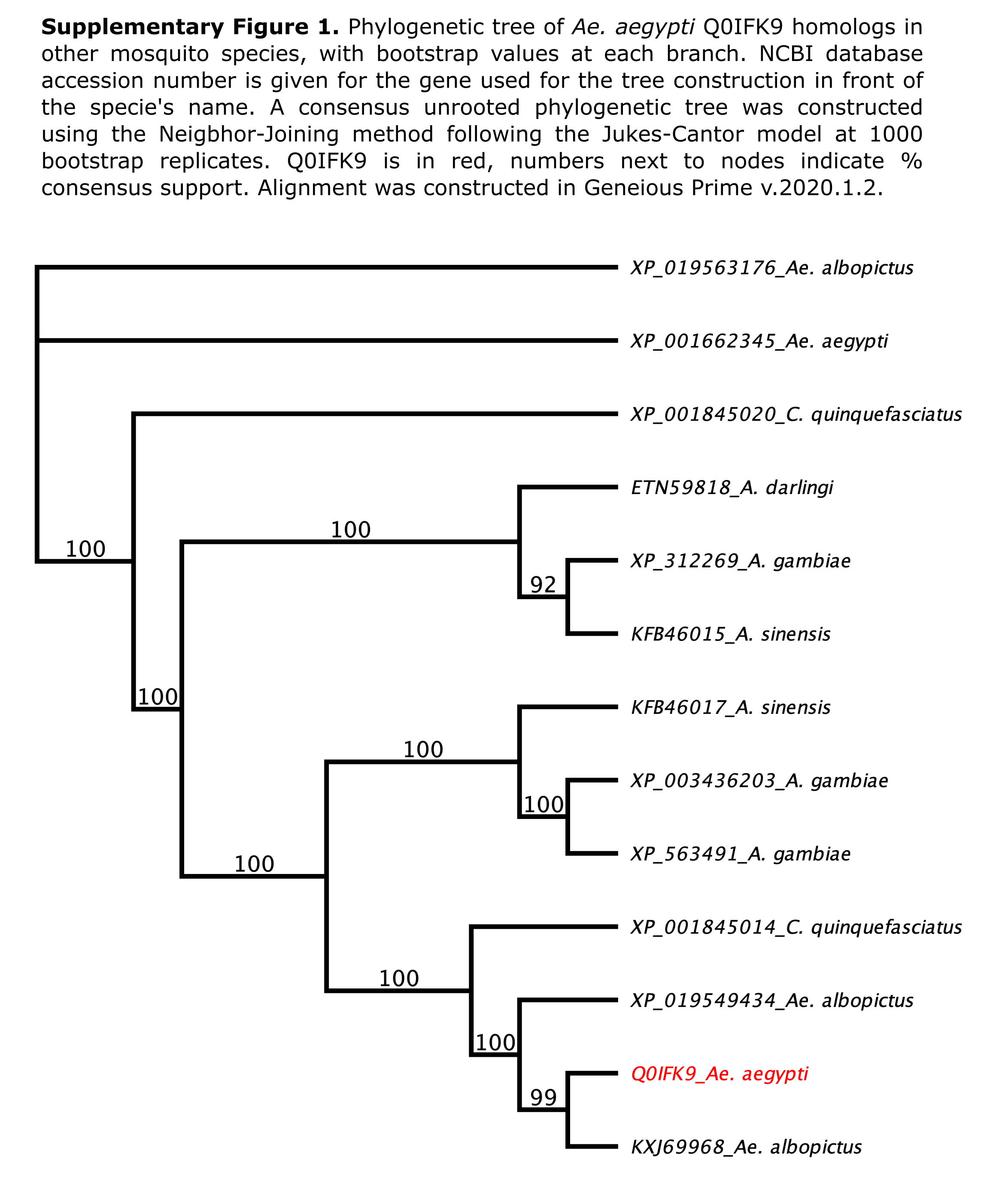

Supplement: Supplementary file 1 [file viruses-12-00748-s001.zip › SFig1.png]
